# Supplementary material for: A blood miRNA signature associates with sporadic Creutzfeldt-Jakob disease diagnosis
Source: Nat Commun. 2020 Aug 7;11:3960. doi: 10.1038/s41467-020-17655-x (PMC7414116; doi:10.1038/s41467-020-17655-x)
Supplement: Supplementary file 1 — Supplementary Information [file 41467_2020_17655_MOESM1_ESM.pdf]

Supplementary Information

A blood miRNA signature associates with sporadic Creutzfeldt-Jakob disease diagnosis

Norsworthy et al.

Supplementary Table 1. Effect of adjustment for RNA Integrity Number (RIN) on small RNA-seq differential expression results.

	**Small RNA-seq Gene Specific Analysis (GSA)**		**RIN <4 excluded (sCJD n=50, Control n=46)[a]**	
miRNA	Adjusted p value[b]	Fold change (sCJD vs. Control)	Adjusted p value	Fold change (sCJD vs. Control)
hsa-miR-93-5p	0.001	-2.34	0.0014	-2.37
hsa-miR-106b-3p	0.008	-1.74	0.005	-1.72
hsa-let-7i-5p	0.011	-2.49	0.02	-2.60
hsa-miR-16-5p	0.0002	-2.76	0.0006	-2.80
hsa-let-7d-3p	0.05	1.94	0.06	2.00

[a]After nine RNA samples with RIN<4 were excluded, 50 biologically independent samples from sCJD patients and 46 biologically independent control samples remained in the analysis; [b]P values were adjusted for multiple testing using the Benjamini-Hochberg false discovery rate (FDR) method.

Supplementary Table 2. **Effect of exclusion of probable sCJD cases on small RNA-seq differential expression results.**

	**Small RNA-seq Gene Specific Analysis (GSA)**		**Probable sCJD cases excluded (sCJD n=54, Control n=48)[a]**	
miRNA	Adjusted p value[b]	Fold change (sCJD vs. Control)	Adjusted p value	Fold change (sCJD vs. Control)
hsa-miR-16-5p	0.00015	-2.76	0.00030	-2.70
hsa-miR-93-5p	0.0010	-2.34	0.00075	-2.33
hsa-miR-106b-3p	0.0080	-1.74	0.011	-1.71
hsa-let-7i-5p	0.011	-2.49	0.011	-2.49
hsa-let-7d-3p	0.053	1.94	0.041	2.00

[a]After three probable sCJD cases were excluded, 54 biologically independent samples from sCJD patients and 48 biologically independent control samples remained in the analysis; [b]P values were adjusted for multiple testing using the Benjamini-Hochberg false discovery rate (FDR) method.

Supplementary Table 3. Partek Gene Specific Analysis of small RNA-seq low abundance miRNAs in samples with >1.5 million raw sequencing reads.

miRNA	Normalised read counts (FPKM[a])	P value (sCJD vs. Control)	Adjusted p value[b] (sCJD vs. Control)	Fold change (sCJD vs. Control)	Least-Square Mean (FPKM, sCJD)	Least-Square Mean (FPKM, Control)
hsa-miR-93-3p	19,026,309	0.002	0.208	-2.393	286,937	683,975
hsa-miR-210-5p	11,744,294	0.010	0.362	-1.163	305,083	352,634
hsa-miR-142-3p	55,305,269	0.018	0.362	1.067	1,569,809	1,468,434
hsa-miR-106b-5p	77,819,291	0.029	0.362	-2.458	1,128,918	2,719,565
hsa-miR-26b-5p	55,512,116	0.036	0.362	-3.177	637,527	1,977,142
hsa-miR-28-5p	12,228,400	0.072	0.448	-1.096	321,120	352,035
hsa-miR-221-3p	29,268,745	0.091	0.448	-1.777	545,098	967,203
hsa-miR-183-5p	33,075,399	0.103	0.448	-1.462	705,323	1,016,590
hsa-miR-181c-5p	16,302,418	0.110	0.448	1.319	531,549	402,741
hsa-miR-19a-3p	35,103,831	0.123	0.448	-1.945	608,359	1,171,798
hsa-miR-339-5p	10,267,082	0.139	0.448	1.152	312,521	271,102
hsa-miR-500a-3p	15,503,149	0.146	0.458	-1.031	423,880	436,764
hsa-miR-1260b	42,088,803	0.161	0.472	1.073	1,244,117	1,157,146
hsa-miR-144-5p	95,295,163	0.189	0.482	-2.487	1,335,329	3,261,340
hsa-miR-27b-3p	56,707,012	0.259	0.491	-2.009	969,075	1,929,345
hsa-miR-30e-5p	37,915,124	0.328	0.550	-1.343	861,914	1,153,170
hsa-miR-1976	14,527,889	0.372	0.596	1.493	506,472	339,144
hsa-miR-23a-3p	33,532,631	0.374	0.596	-1.145	861,087	985,567
hsa-miR-550a-5p	12,776,984	0.411	0.639	1.131	384,628	339,915
hsa-miR-502-3p	13,734,181	0.446	0.656	1.079	399,758	369,950
hsa-miR-342-5p	13,566,018	0.448	0.656	1.033	387,899	375,119
hsa-miR-1249-3p	18,137,643	0.501	0.694	-1.010	512,751	514,136
hsa-miR-143-3p	20,608,162	0.541	0.722	-1.603	398,703	630,212
hsa-miR-2110	17,320,059	0.549	0.722	1.521	613,304	403,376
hsa-miR-335-5p	15,800,179	0.553	0.722	1.417	542,188	382,142
hsa-miR-3613-3p	14,478,723	0.578	0.731	1.422	496,186	348,861
hsa-miR-769-5p	13,202,045	0.602	0.745	1.043	377,810	362,122
hsa-miR-21-5p	87,021,208	0.613	0.745	-2.769	1,116,679	3,028,884
hsa-miR-5010-3p	13,482,171	0.668	0.763	1.260	431,193	341,988
hsa-miR-664a-3p	15,747,928	0.815	0.869	-1.077	418,770	450,884

[a]Fragments Per Kilobase of transcript per Million mapped reads; [b]P values were adjusted for multiple testing using the Benjamini-Hochberg false discovery rate (FDR) method.

Supplementary Table 4. Identification of DE miRNA Targets for qPCR using TarBase[58] and GTEx[55].

DE miRNA	Target gene	Validation assay type/s[a]	No. cell lines	No. tissues	No. publications	Prediction score (miTG score[b])	GTEx expression in whole blood[55] (Median TPM[c])
hsa-miR-16-5p	*CCND3*	LC, IP, MA	5	5	4	0.917	133.5
	VEGFA	LC, WB, qPCR, IP, MA	8	3	10	-	14.73
hsa-miR-93-5p	*VEGFA*	LC, IP	2	1	2	-	14.73
	CDKN1A	LC, IP	7	5	9	0.942	23.13
hsa-let-7i-5p	*ZFP36*	IP	5	4	6	-	541.5
	NAP1L1	IP	7	5	6	-	12.98
	RNF44	IP	7	5	9	-	33.23

[a]LC (luciferase reporter assay), IP (immunoprecipitation), MA (Microarray), WB (Western Blot), qPCR (quantitative PCR); [b]The higher the miTG score the higher the probability of targeting; [c]Transcripts per million.

Supplementary Table 5. Clinical and sampling details for sCJD patients in the longitudinal cohort.

Patient no.	No. PAX blood samples	Sample intervals (days)	Final diagnosis	Sex	PRNP codon 129 genotype	Age at onset	Presentation type	Clinical category	Baseline MRC Scale score[56]	MRC Scale slope[4]	Disease duration (days)	Magnetic Resonance Imaging (MRI) finding	Cerebrospinal Fluid (CSF) Protein 14-3-3	CSF RT-QuIC[a]	Electroencephalogram (EEG) finding
1	2	22	Probable sCJD	Male	VV	70	Ataxia	Classical	12	0.628	122	Small vessel disease	Positive	No data	No data
2	2	204	Definite sCJD	Male	MV	58	Paranoia	Neuropsychiatric	13	0.198	566	Cortical ribboning	Positive	No data	Slow
3	2	19	Definite sCJD	Male	VV	79	Blurred vision	Classical	6	0.886	137	Movement artefact, no CJD features	No data	No data	Slow
4	2	46	Probable sCJD	Male	MV	68	Tremor and ataxia	Classical	6	0.187	358	No data	Positive	No data	Slow
5	4	36,96,37	Definite sCJD	Male	MV	63	Memory and attention deficit	Cognitive	17	0.261	835	Basal ganglia restriction, cortical ribboning	No data	No data	Semi-periodic complexes
6	2	70	Definite sCJD	Male	MV	52	Ataxia	Ataxic	13	0.291	387	Basal ganglia and thalamic restriction, cortical ribboning	Positive	No data	Slow
7	4	40,78,118	Definite sCJD	Female	MV	54	Ataxia	Ataxic	5	0.085	947	Basal ganglia restriction, cortical ribboning	Positive	No data	Slow
8	2	139	Definite sCJD	Female	MV	57	Episodic memory decline	Cognitive	14	0.045	1930	Basal ganglia and thalamic restriction, cortical ribboning	Positive	No data	Slow
9	4	57,62,57	Probable sCJD	Female	MV	57	Ataxia	Ataxic	8	0.135	942	Basal ganglia restriction, cortical ribboning	No data	No data	Slow
10	2	30	Probable sCJD	Male	VV	74	Personality change	Classical	11	0.642	124	Small vessel disease	Positive	No data	Slow
11	3	102,164	Definite sCJD	Female	MV	65	Insomnia	Sleep/thalamic	19	0.203	564	Cortical ribboning	Positive	No data	Slow

Patient no.	No. PAX blood samples	Sample intervals (days)	Final diagnosis	Sex	PRNP codon 129 genotype	Age at onset	Presentation type	Clinical category	Baseline MRC Scale score[56]	MRC Scale slope[4]	Disease duration (days)	Magnetic Resonance Imaging (MRI) finding	Cerebrospinal Fluid (CSF) Protein 14-3-3	CSF RT-QuIC[a]	Electroencephalogram (EEG) finding
12	2	27	Probable sCJD	Female	VV	53	Ataxia	Ataxic	14	0.761	212	Basal ganglia and thalamic restriction, cortical ribboning	Positive	No data	Slow
13	2	91	Definite sCJD	Male	MM	70	Planning and memory decline	Cognitive	14	0.353	245	Cortical ribboning	Positive	No data	Slow
14	3	62,70	Definite sCJD	Female	MV	72	Memory decline	Classical	13	0.182	263	Basal ganglia and thalamic restriction, cortical ribboning	Negative	Positive	Normal
15	2	148	Probable sCJD	Male	MV	55	Personality change	Classical	13	0.167	472	Basal ganglia and thalamic restriction, cortical ribboning	Negative	Negative	No data
16	3	25,27	Definite sCJD	Female	MM	51	Memory decline	Cognitive	14	1.085	157	Basal ganglia restriction, cortical ribboning	Positive	No data	Normal
17	2	42	Probable sCJD	Female	MM	69	Patchy visual field loss	Visual	11	0.978	306	Normal	Positive	No data	Slow
18	2	38	Definite sCJD	Male	VV	50	Memory decline	Cognitive	18	0.943	374	Basal ganglia restriction, cortical ribboning	Positive	No data	Slow
19	2	41	Definite sCJD	Male	VV	65	Ataxia and diplopia	Classical	13	0.883	236	Cortical ribboning	Positive	Positive	Slow
20	2	27	Probable sCJD	Male	VV	59	Insomnia	Sleep/thalamic	13	0.755	229	Basal ganglia and thalamic restriction, cortical ribboning	No data	No data	Slow
21	2	17	Definite sCJD	Male	MV	58	Ataxia	Ataxia	14	0.551	254	Basal ganglia restriction	No data	No data	No data

[a]Real Time Quaking-induced Conversion

Supplementary Table 6. **Summary data for Area Under Curve – Receiver Operating Characteristics (AUC-ROC) curve analysis.**

Comparison	microRNA[a]	AUC	Maximum Youden's Index	Z Score threshold[b]	Sensitivity (%)	Specificity (%)
**sCJD vs Control (Discovery cohort)**	hsa-miR-16-5p	0.762	0.495	0.241	80.7	68.8
	hsa-miR-93-5p	0.760	0.446	0.349	84.2	60.4
	hsa-let-7i-5p	0.736	0.451	0.680	93.0	52.1
	Aggregated	0.788	0.568	0.140	86.0	70.8
**sCJD vs Control (Replication cohort)**	hsa-miR-16-5p	0.652	0.318	-0.491	55.2	76.7
	hsa-miR-93-5p	0.652	0.320	-0.142	58.6	73.3
	hsa-let-7i-5p	0.668	0.315	-0.481	44.8	86.7
	Aggregated	0.667	0.321	-0.192	62.1	70.0
**AD vs Control**	hsa-miR-16-5p	0.859	0.633	0.008	86.7	76.7
	hsa-miR-93-5p	0.810	0.533	-0.623	96.7	56.7
	hsa-let-7i-5p	0.851	0.567	0.043	76.7	80.0
	hsa-let-7i-5p	0.851	0.567	-0.242	86.7	70.0
	hsa-let-7i-5p	0.851	0.567	0.186	73.3	83.3
	Aggregated	0.860	0.567	0.131	80.0	76.7
	Aggregated	0.860	0.567	-0.227	90.0	66.7
	Aggregated	0.860	0.567	-0.0001	83.3	73.3
**sCJD vs AD**	hsa-miR-16-5p	0.921	0.763	0.254	89.7	86.7
	hsa-miR-93-5p	0.897	0.694	0.043	82.8	86.7
	hsa-let-7i-5p	0.934	0.725	-0.219	75.9	96.7
	Aggregated	0.924	0.724	-0.306	72.4	100.0

[a]ROC curves were plotted using Z scores generated from log transformed expression data for each miRNA individually, and also aggregated by calculating the mean Z score of hsa-miR-16-5p, hsa-miR-93-5p and hsa-let-7i-5p; [b]For sCJD comparisons, positive test results are below the threshold, for AD vs Control, positive test results are above the threshold.

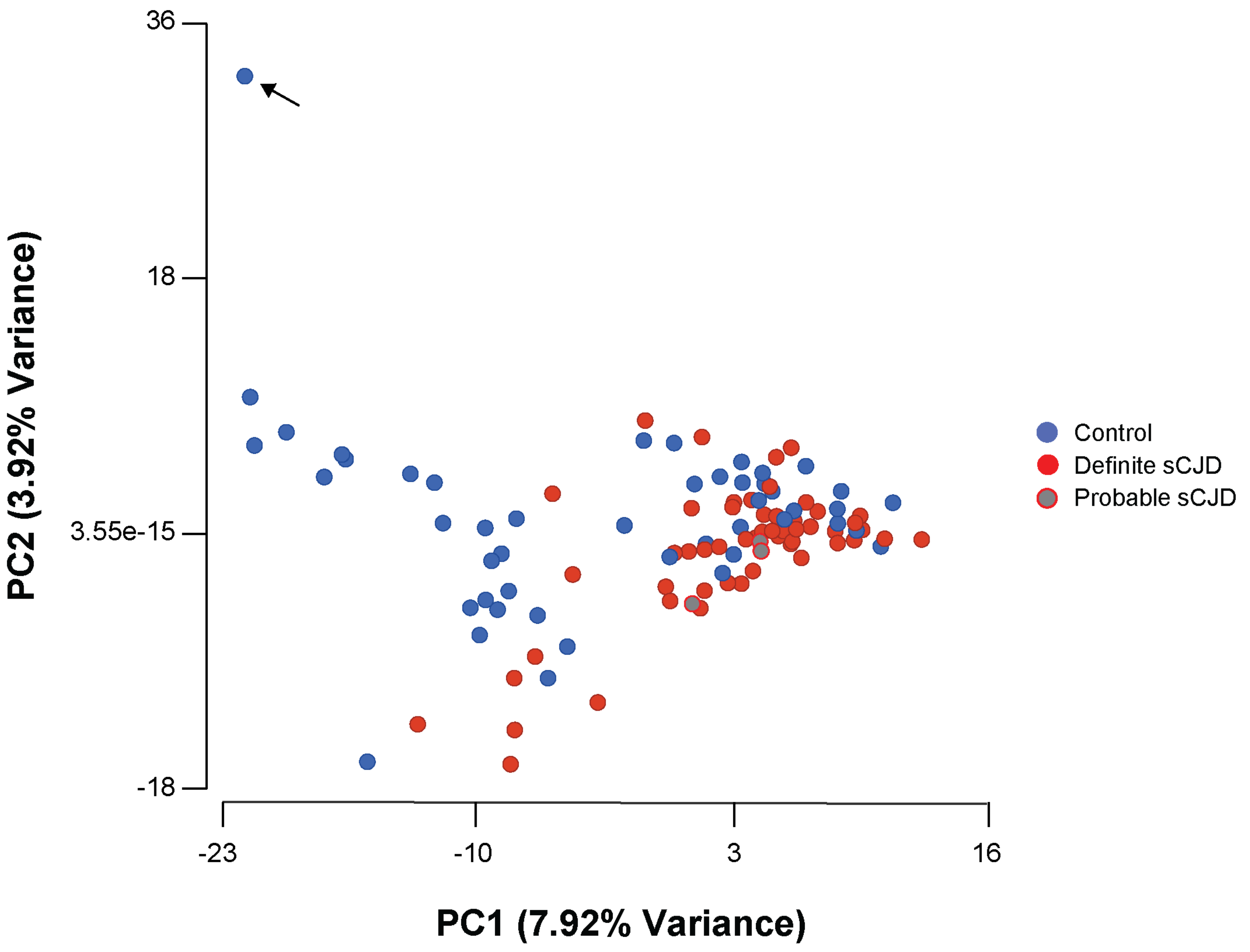

Supplementary Fig. 1. PCA plot based on normalised read counts from all samples in the discovery cohort. The first two principal components are shown. The outlier control sample (indicated with arrow in top left hand corner) did not stand out from other controls in terms of metadata or sequencing metrics. Definite sCJD (red) n=54; Probable sCJD (grey with red outline) n=3; Controls (blue) n=48.

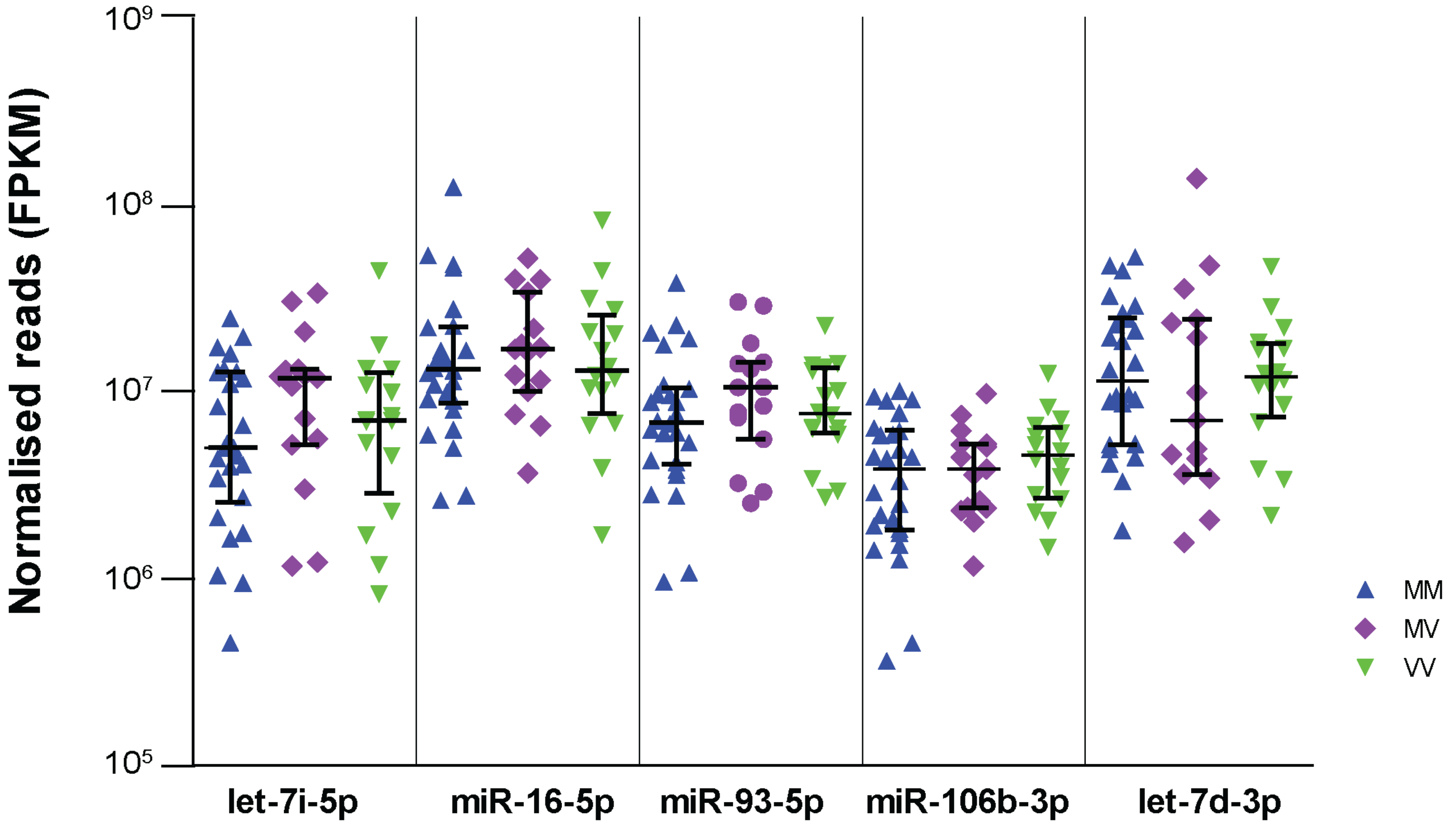

Supplementary Fig. 2. *PRNP* codon 129 genotype and expression of differentially expressed (DE) miRNAs in discovery cohort sCJD patients. Normalised transcript counts (in Fragments Per Kilobase of transcript per Million mapped reads, FPKM) were plotted with respect to PRNP codon 129 genotype (MM n=26, MV n=15, VV n=16). Median and interquartile range are shown. Source data are provided as a Source Data file.

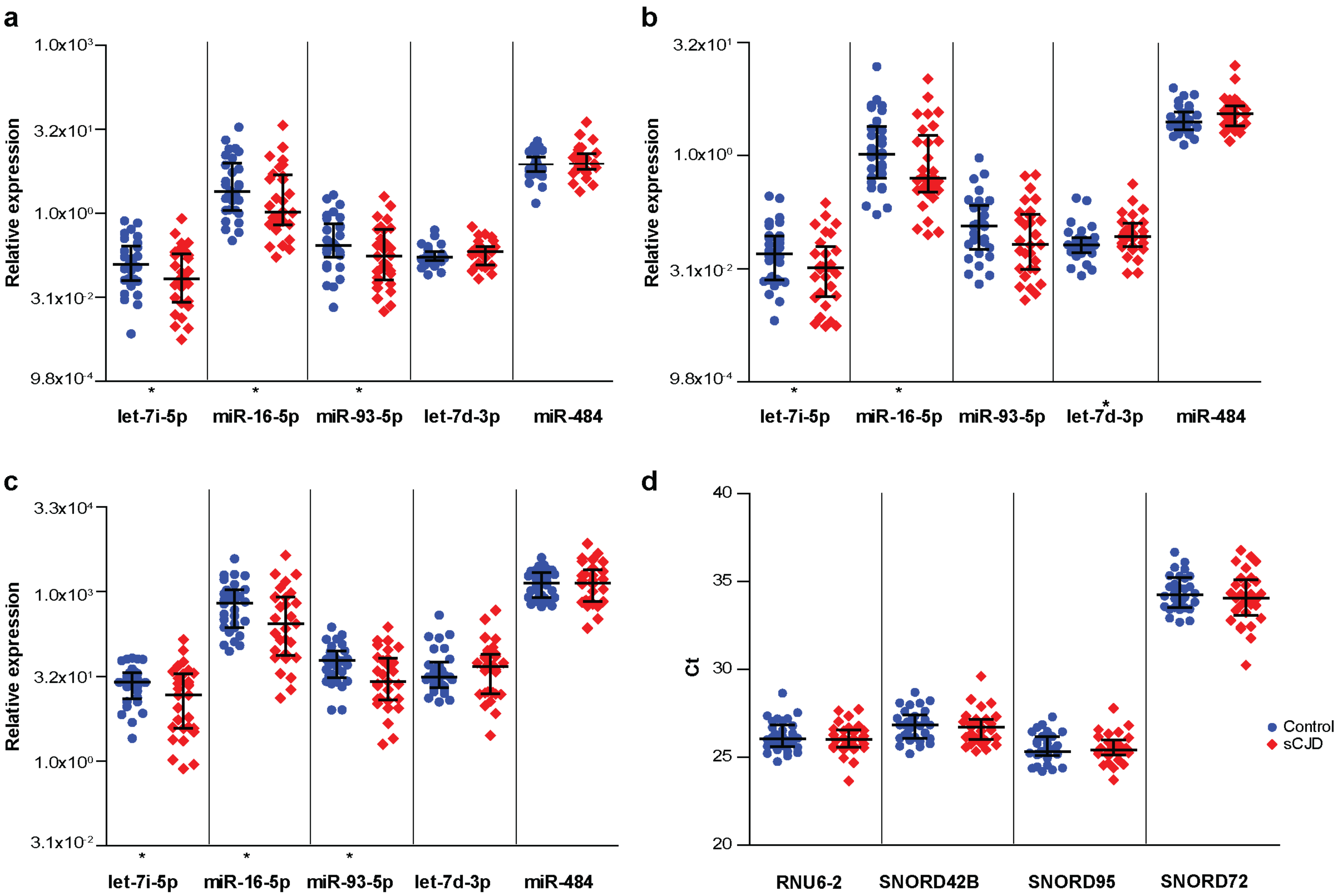

Supplementary Fig. 3. Validation and replication of DE miRNAs measured by qRT-PCR. Expression of DE miRNAs relative to the control small RNA SNORD42B (**a**) hsa-let-7i-5p Fold change (FC) -1.75, p=0.048; hsa-miR-16-5p FC -1.82, p=0.039; hsa-miR-93-5p FC -1.70, p=0.043; hsa-let-7d-3p FC 1.14, p=0.089; hsa-miR-484 FC 1.13, p=0.270; SNORD95 (**b**) hsa-let-7i-5p FC -1.64, p=0.041; hsa-miR-16-5p FC -1.65, p=0.046; hsa-miR-93-5p FC -1.54, p=0.074; hsa-let-7d-3p FC 1.29, p=0.026; hsa-miR-484 FC 1.21, p=0.060; and SNORD72 (**c**) hsa-let-7i-5p FC -1.99, p=0.032; hsa-miR-16-5p FC -2.07, p=0.030; hsa-miR-93-5p FC -1.93, p=0.016; hsa-let-7d-3p FC 1.05, p=0.342; hsa-miR-484 FC 1.00, p=0.473; with median and interquartile range, are shown for both control and sCJD groups. One-sided Mann-Whitney tests were used to generate p-values. Raw Ct values (**d**) were plotted for all four control small RNAs, and are shown with median and interquartile range in both control and sCJD groups. Control (blue circles) n=30 biologically independent samples; sCJD (red diamonds) n=29 biologically independent samples. *p<0.05. Source data for (**a-d**) are provided as a Source Data file.

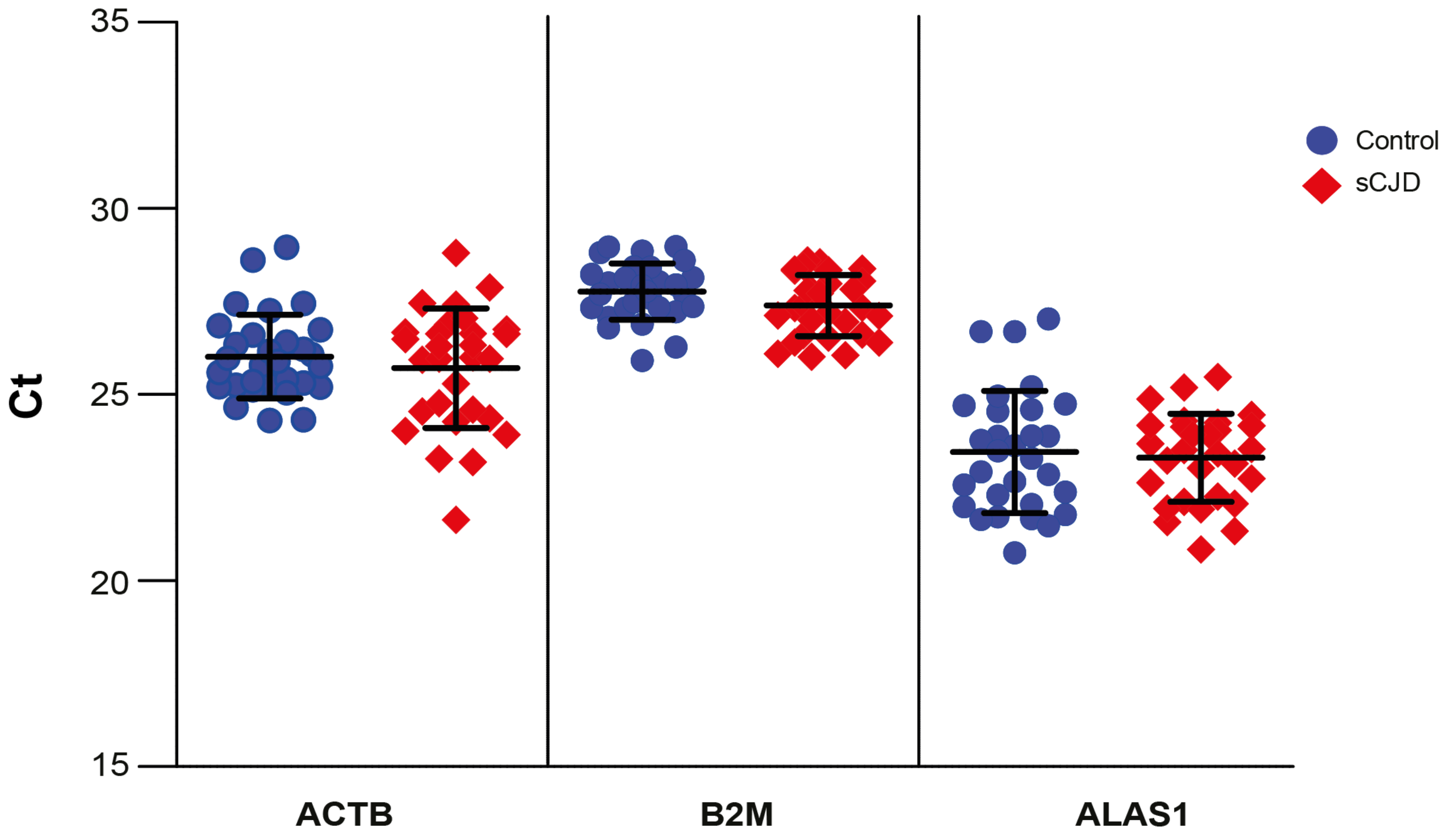

Supplementary Fig. 4. Raw Ct values in control and sCJD groups for mRNA target endogenous control RNAs. Ct values were plotted for all three endogenous control RNAs (shown on x axis) and are shown with mean and standard deviation in both control and sCJD groups. sCJD n=29 biologically independent samples; controls n=30 biologically independent samples. Source data are provided as a Source Data file.

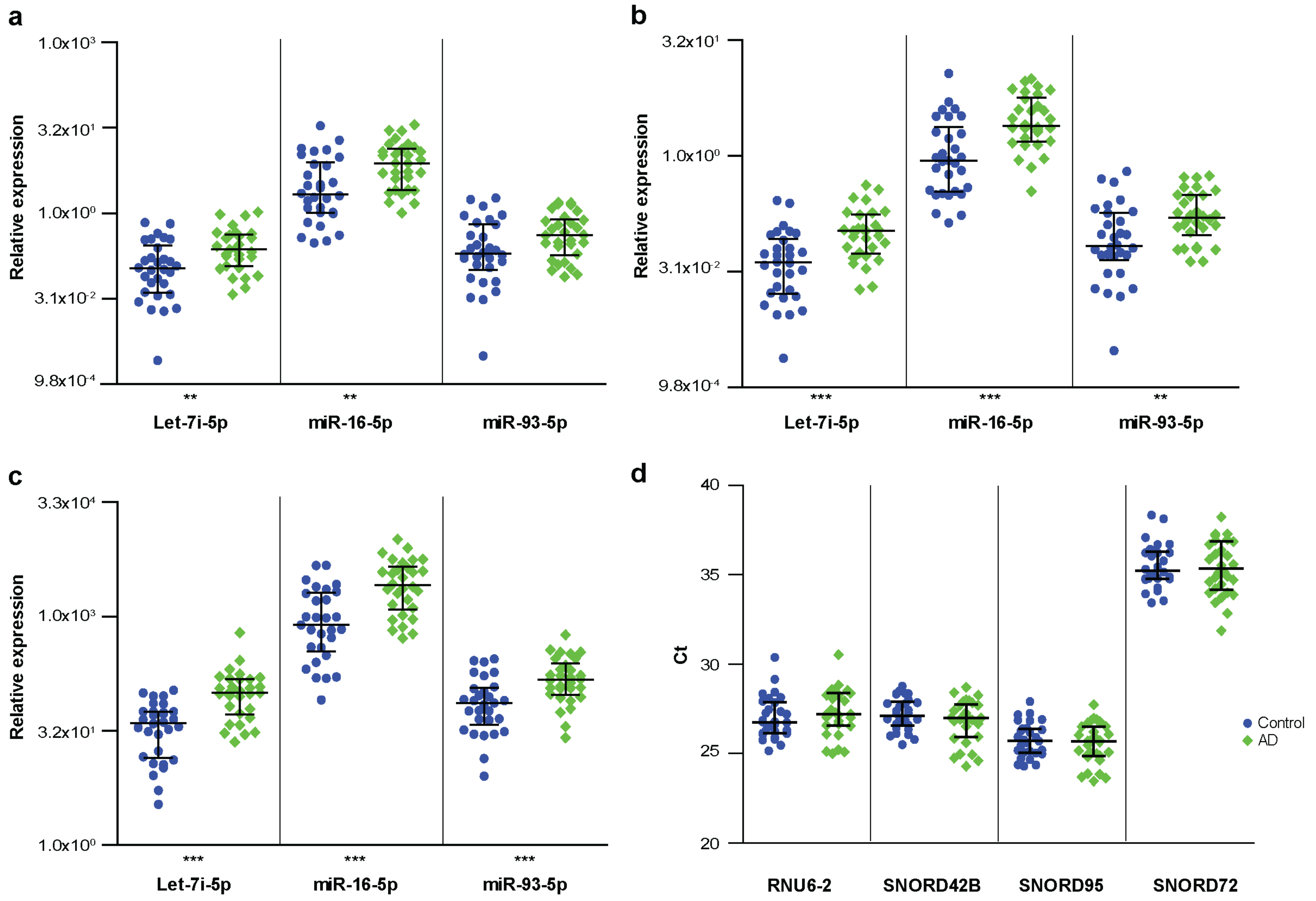

Supplementary Fig. 5. Expression of replicated DE miRNAs in AD measured by qRT-PCR. Expression of DE miRNAs relative to the control small RNA SNORD42B (**a**), hsa-let-7i-5p FC 2.36, $p=3.2x10^{-3}$; hsa-miR-16-5p FC 2.69, $p=2.9x10^{-3}$; hsa-miR-93-5p FC 1.94, p=0.050; SNORD95 (**b**) hsa-let-7i-5p FC 2.57, $p=2.8x10^{-4}$; hsa-miR-16-5p FC 2.96, $p=2.5x10^{-4}$; hsa-miR-93-5p FC 2.14, $p=7.6x10^{-3}$; and SNORD72 (**c**) hsa-let-7i-5p FC 2.91, $p=1.8x10^{-5}$; hsa-miR-16-5p FC 3.15, $p=1.3x10^{-4}$; hsa-miR-93-5p FC 2.12, $p=6.8x10^{-4}$; with median and interquartile range, are shown for both control and AD groups. Two-sided Mann-Whitney tests were used to generate p-values. Raw Ct values (**d**) were plotted for all four control small RNAs, and are shown with median and interquartile range in both control and AD groups. Control (blue circles) n=30 biologically independent samples; AD (green diamonds) n=30 biologically independent samples. **p<0.01 ***p<0.001. Source data for (**a-d**) are provided as a Source Data file.

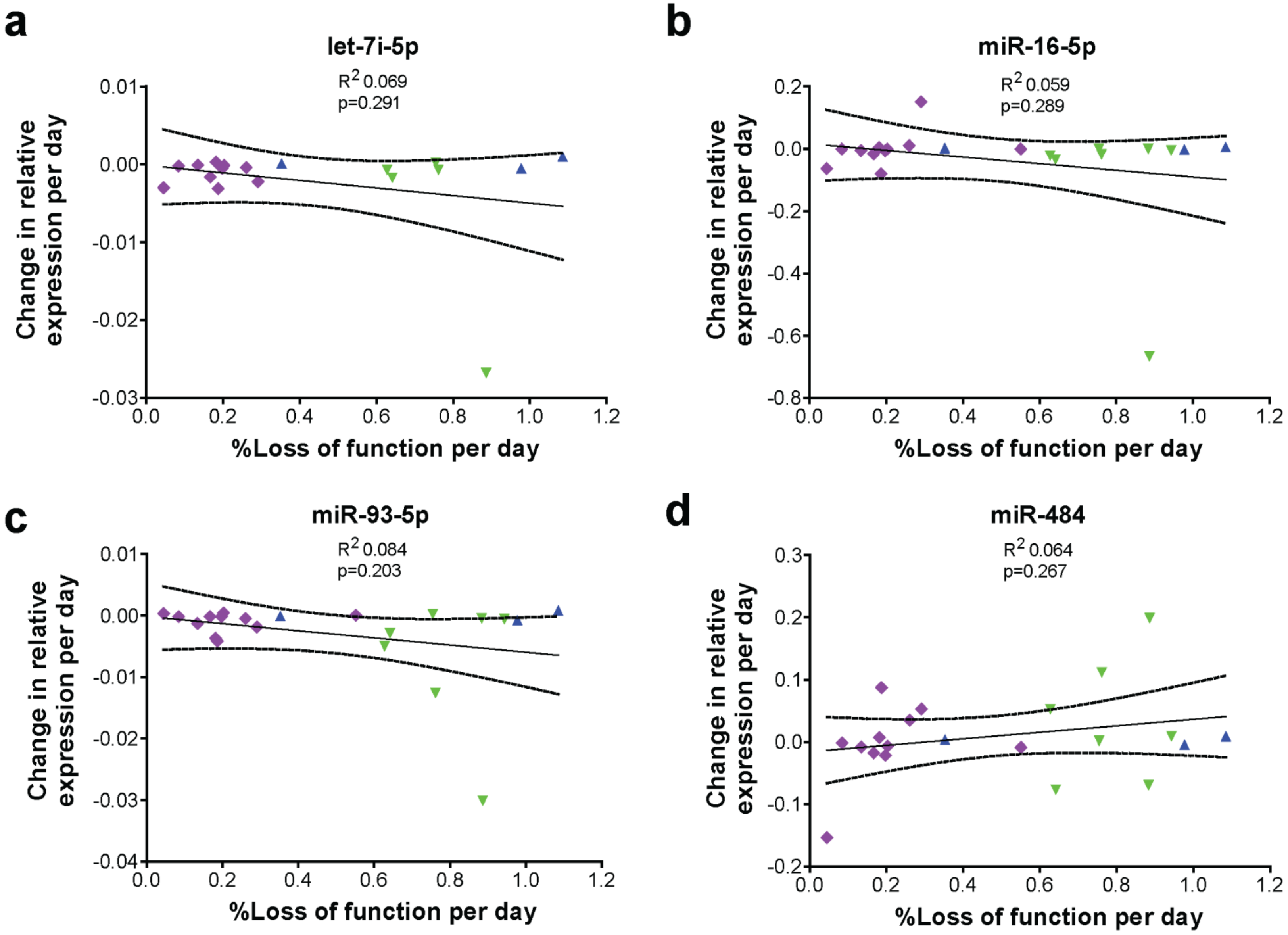

Supplementary Fig. 6. Relationship between disease progression and change in DE miRNA levels in longitudinal samples from sCJD patients (control RNA SNORD42B). miRNA expression was measured relative to the control small RNA SNORD42B for (**a**) hsa-let-7i-5p, (**b**) hsa-miR-16-5p, (**c**) hsa-miR-93-5p and (**d**) hsa-miR-484. hsa-miR-484 is a non-DE microRNA shown here for comparison. %Loss of function per day was calculated from decline in MRC Scale Score. Linear regression R^2 and p values are shown. Dotted lines represent 95% confidence intervals. Data points are colour coded by *PRNP* codon 129 genotype (MM blue triangle, MV magenta diamonds, VV inverted green triangles). n=21 individuals (18 for let-7i-5p). Source data for (**a-d**) are provided as a Source Data file.

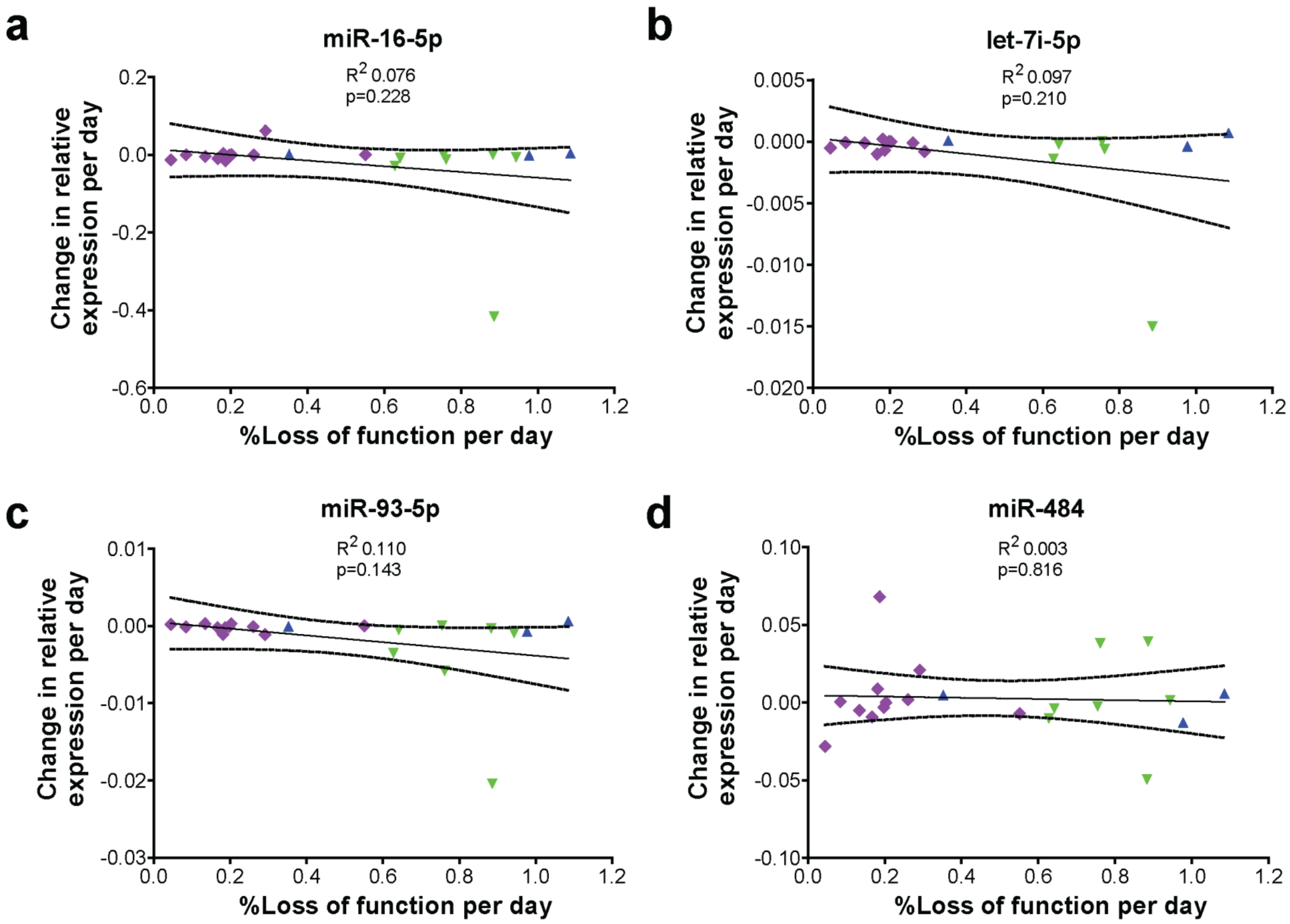

Supplementary Fig. 7. Relationship between disease progression and change in DE miRNA levels in longitudinal samples from sCJD patients (control RNA SNORD95). miRNA expression was measured relative to the control small RNA SNORD95 for (**a**) hsa-miR-16-5p,(**b**) hsa-let-7i-5p, (**c**) hsa-miR-93-5p and (**d**) hsa-miR-484. hsa-miR-484 is a non-DE microRNA shown here for comparison. %Loss of function per day was calculated from decline in MRC Scale Score. Linear regression R^2 and p values are shown. Dotted lines represent 95% confidence intervals. Data points are colour coded by *PRNP* codon 129 genotype (MM blue triangle, MV magenta diamonds, VV inverted green triangles). n=21 individuals (18 for let-7i-5p). Source data for (**a-d**) are provided as a Source Data file.

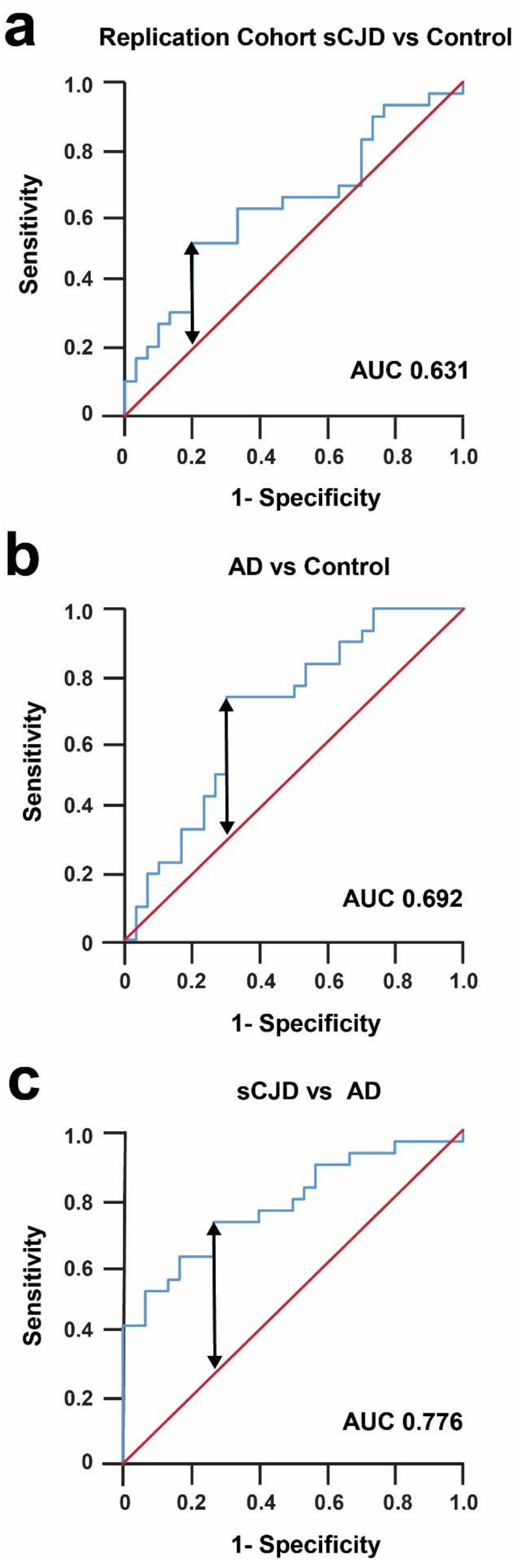

Supplementary Fig. 8 Area Under Curve – Receiver Operating Characteristic (AUC-ROC) plots derived from DE miRNA qRT-PCR data (control RNA SNORD42B). These were plotted using mean Z scores calculated from log transformed ratios of hsa-let-7i-5p, hsa-miR-16-5p and hsa-miR-93-5p measured relative to SNORD42B for (**a**) sCJD patients and control individuals, sCJD n=29 biologically independent samples; control n=30 biologically independent samples (**b**) AD patients and control individuals, AD n=30 biologically independent samples; control n=30 biologically independent samples and (**c**) sCJD and AD patients, sCJD n=29 biologically independent samples; AD n=30 biologically independent samples. The line of zero discrimination is shown in red. AUC value is shown, and maximal Youden's Index is indicated by the double headed arrow, where test performance is optimal. Source data for (**a-c**) are provided as a Source Data file.

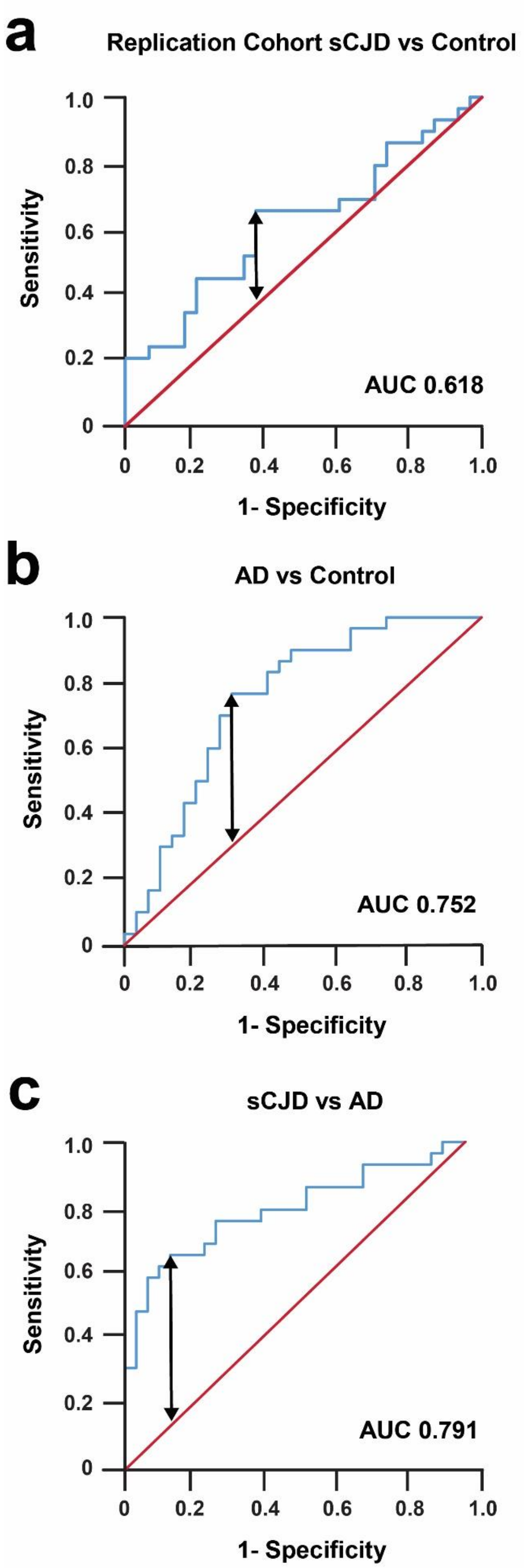

Supplementary Fig. 9. Area Under Curve – Receiver Operating Characteristic (AUC-ROC) plots derived from DE miRNA qRT-PCR data (control RNA SNORD95). These were plotted using mean Z scores calculated from log transformed ratios of hsa-let-7i-5p, hsa-miR-16-5p and hsa-miR-93-5p measured relative to SNORD95 for (**a**) sCJD patients and control individuals, sCJD n=29 biologically independent samples; control n=30 biologically independent samples (**b**) AD patients and control individuals, AD n=30 biologically independent samples; control n=30 biologically independent samples and (**c**) sCJD and AD patients, sCJD n=29 biologically independent samples; control n=30 biologically independent samples. The line of zero discrimination is shown in red. AUC value is shown, and maximal Youden's Index is indicated by the double headed arrow, where test performance is optimal. Source data for (**a-c**) are provided as a Source Data file.

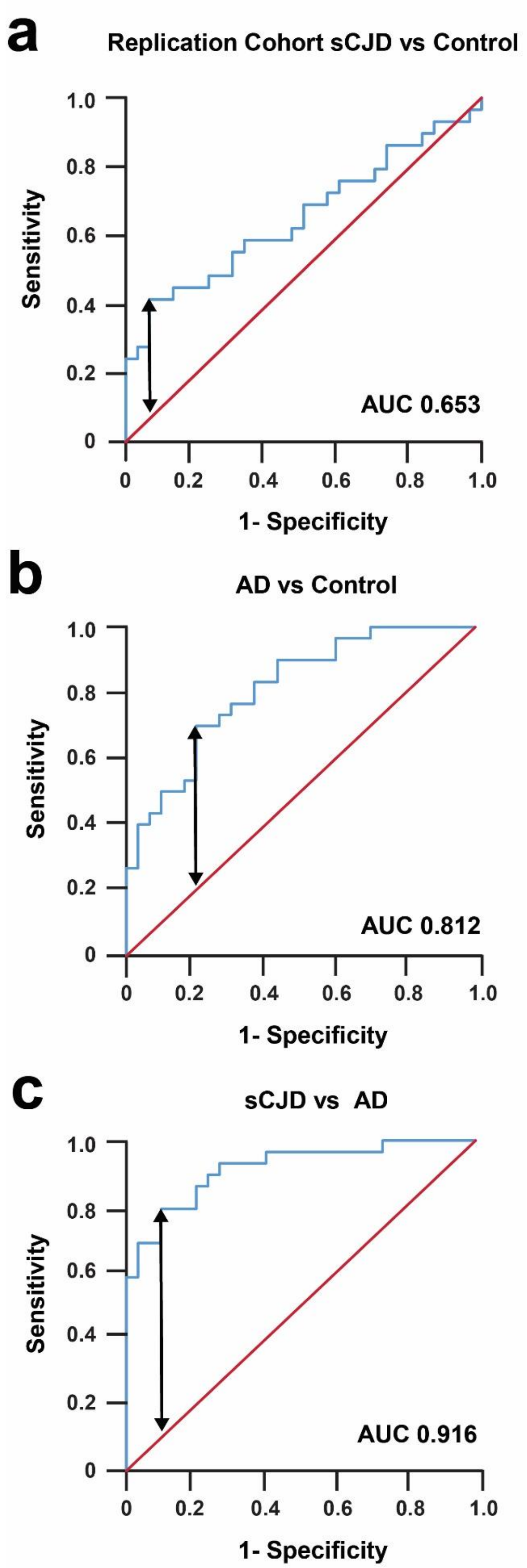

Supplementary Fig. 10. Area Under Curve – Receiver Operating Characteristic (AUC-ROC) plots derived from DE miRNA qRT-PCR data (control RNA SNORD72). These were plotted using mean Z scores calculated from log transformed ratios of hsa-let-7i-5p, hsa-miR-16-5p and hsa-miR-93-5p measured relative to SNORD72 for (**a**) sCJD patients and control individuals, sCJD n=29 biologically independent samples; control n=30 biologically independent samples (**b**) AD patients and control individuals, AD n=30 biologically independent samples; control n=30 biologically independent samples and (**c**) sCJD and AD patients, sCJD n=29 biologically independent samples; AD n=30 biologically independent samples. The line of zero discrimination is shown in red. AUC value is shown, and maximal Youden's Index is indicated by the double headed arrow, where test performance is optimal. Source data for (**a-c**) are provided as a Source Data file.

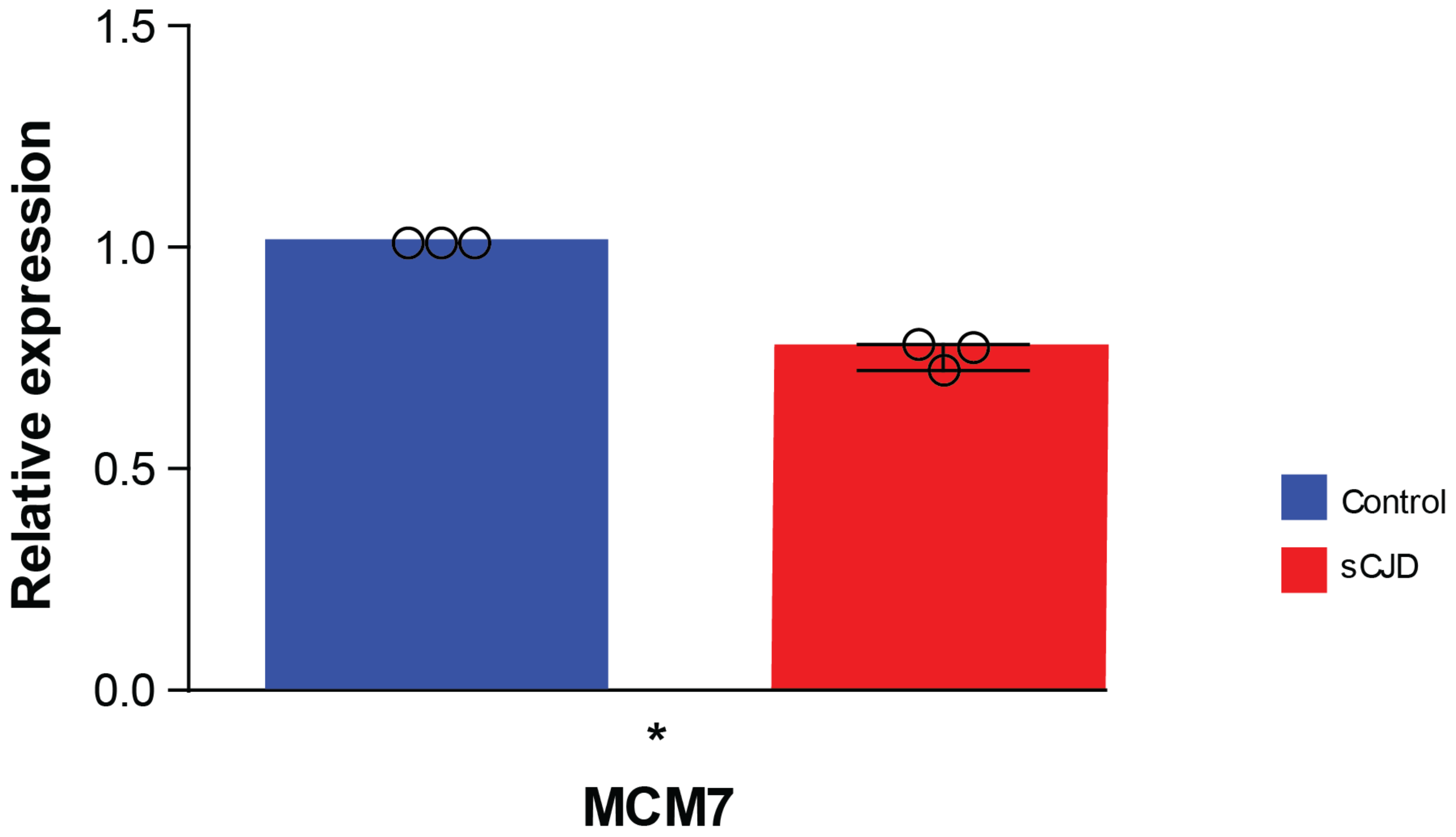

Supplementary Fig. 11. Expression of *MCM7* in replication cohort controls and sCJD patients. Combined expression of *MCM7* plotted relative to combined expression of control mRNAs (*ALAS1*, *B2M* and *ACTB*). Median and range are shown with individual data points (black open circles). Median FC is -1.30 in sCJD patients compared to controls, p=0.032 (one-sided Mann-Whitney test). sCJD n=29 biologically independent samples; controls n=30 biologically independent samples.*p <0.05. Source data are provided as a Source Data file.
