# Supplementary material for: A blood miRNA signature associates with sporadic Creutzfeldt-Jakob disease diagnosis
Source: Nat Commun. 2020 Aug 7;11:3960. doi: 10.1038/s41467-020-17655-x (PMC7414116; doi:10.1038/s41467-020-17655-x)
Supplement: Supplementary file 2 — Peer Review File [file 41467_2020_17655_MOESM2_ESM.docx]

Peer Review Information

**Journal:** Nature Communications

**Manuscript title: A blood miRNA signature associates with sporadic Creutzfeldt-Jakob disease diagnosis and rate of progression**

**Corresponding author name(s):** Prof Simon Mead

**Editorial notes:**

**none**

**Reviewer comments & decisions:**

**Reviewer comments, first version:**

Reviewer #1 (Remarks to the Author):

In this study, a signature of 5 altered microRNAs (miRNAs) in blood was identified in a cohort of sporadic Creutzfeldt-Jakob disease (sCJD) patients using small RNA-sequencing. The decreased abundance of 3/5 miRNAs were further replicated in an independent sCJD cohort using quantitative PCR (qPCR). Additionally, increased abundance in blood of 4 target genes of the miRNA signature was also detected in the sCJD patients. By measuring the expression of the miRNA signature in Alzheimer’s disease (AD) patients it was also revealed that this signature can discriminate between sCJD and AD. Finally, by longitudinally measuring miRNA expression in a third sCJD cohort, the rate of decline for the 3 miRNAs was compared to the rate of sCJD disease progression. This study strongly benefits from a careful experimental design and relatively large numbers of individual samples, which can be difficult to achieve for sCJD due to the rarity of this disorder.
It is of interest to others in the prion field as it is the first to provide a robust analysis of circulating miRNAs in a human prion disease, although one study of serum miRNAs in chronic wasting disease, a prion disease of deer was recently published (https://doi.org/10.1038/s41598-019-56249-6). Statistical analysis is appropriate and for the most part there is sufficient detail for a researcher to reproduce the work.

Specific comments:

1. In this manuscript miRNA profiling is performed in whole blood rather than serum or plasma. This has some advantages such as it is less prone to the variations caused by sample manipulation, results are not biased by cell lysis and it is more amenable to normalization using commonly used housekeeping genes.

However, there are also some disadvantages that have not been discussed in the manuscript. Extremely high levels of a number of miRNAs are associated with red blood cells (RBCs) for example. When using a direct ‘count’ based strategy such as NGS this means that changes in less abundant miRNAs tend to be masked, thus concealing disease specific patterns. This should be mentioned in relation to the relatively limited correlation found with other studies of altered miRNA abundance in prion diseases.

A second disadvantage that is not mentioned in the manuscript is miRNAs are expressed in varying amounts in the different cell types found in blood. (e.g. https://doi.org/10.1016/j.gpb.2019.09.004). The authors should comment on biological variation in cell counts in relation to disease and disease progression and how that may affect the miRNA profiles obtained. Are the authors able to associate cell counts with miRNA abundance? Are cell counts available for the patients in the study and can this data be used in a normalization step for the data? If so could the authors reanalyze the data to account for, for example, RBC contribution to the profiles. It is possible that more disease related profiles could be determined by performing some further normalization steps.

Accordingly miR-16-5p, identified in this study as a significant disease-associated miRNA, is extremely highly expressed in red blood cells. The authors comment on the importance of this miRNA to prion disease in reference to studies on infected brain tissue. Is it possible that altered abundance in the blood is unrelated to the mechanism by which it is deregulated in neurons or neurodegeneration specifically?

2. It is mentioned that 1 million reads was the cut-off in these analyses. Given that a small number of miRNAs make up the majority of read counts in blood this is likely a suboptimal number of reads for this type of study. If some samples resulted in significantly more reads than others perhaps the authors could comment on, or provide an analysis of some of the lower abundance miRNAs if they can be identified in these samples.

3. The very rapid decline in sCJD would likely mean that these patients would only correlate to AD patients very late in their generally much longer disease course. When comparing AD and sCJD samples confirm that the disease stage is equivalent.

4. In figure 4 a significant relationship was observed between the change in relative miRNA expression per day and %loss of function per day. However, it appears from the graphs that a single sample with the fastest decline in miRNA expression and fastest rate of disease progression is driving statistical significance, with the remaining samples showing little correlation between miRNA expression and disease progression. The claim that miRNA expression associates with disease progression should be strengthened by addressing if this sample is an outlier (is statistical significance retained if the sample is removed?) or if any confounding factors could have influenced miRNA abundance in this sample. It is also possible that the relationship is non-linear. Additionally, no information was provided regarding the timing of the longitudinal sampling (i.e. how long between each sampling). This should be included to provide more information about how the rate of miRNA decline was calculated, perhaps as a Supplemental table or figure.

5. A recent publication that showed increased let-7i-5p abundance in serum from elk with chronic wasting disease and decreased miR-16-5p abundance in serum from scrapie infected hamsters was not referenced. See:
Slota JA, Medina SJ, Klassen M, et al. Identification of circulating microRNA signatures as potential biomarkers in the serum of elk infected with chronic wasting disease. Sci Rep. 2019;9(1):19705. Published 2019 Dec 23. doi:10.1038/s41598-019-56249-6
In this manuscript greater correlation between serum miRNA profiles in a prion disease and neurodegeneration (AD) was reported than seen here. Do the authors think that this may be evidence of masking of disease related patterns by the inclusion of red blood cell miRNAs in the analysis in this case?

6. Clarify that the RIN numbers were those of total RNA rather than the small RNA fraction.

7. Why were the read counts expressed as FPKM as this is generally used in RNA Seq to normalize paired end reads with the varying lengths of transcripts. Here the experiment is designed to capture only reads of mature fully processed miRNAs 15-25bp.

8. Clarify the method of normalization of the read data – for example, global mean normalization scaling?

9. It is encouraged to make datasets such as this publicly available by submitting to databases such as NCBI’s GEO.

10. It would be beneficial to see the inclusion of PCA plots based on analysis of the total data. This is a useful way for visual quality control. This could be included as a figure/supplementary data to clearly illustrate the relationship between samples and to highlight any outlier samples.


Reviewer #2 (Remarks to the Author):

The paper by Norsworthy and co-workers investigates the role of blood microRNAs (miRNAs) in patients diagnosed with sporadic Creutzfeldt-Jakob disease (sCJD) and their feasibility as blood-based diagnostic assay. The clinical diagnosis of sCJD is usually based on the combination of a neuropsychiatric syndrome and presence of pathologically aggregated prion protein in an aggregation assay done on the patient’s cerebrospinal fluid. Obtaining CSF by lumbar puncture is not without risks such as post-puncture headaches, bleeding, infection. Here, a blood-based assay for the diagnosis of sCJD could represent a substantial improvement. Additionally, majority of sCJD research has been focused on the central nervous system while changes in peripheral organs are widely unclear.
The paper is well-written and adequately powered considering the rare incidence of sCJD. The authors show that a subset of miRNAs is differentially expressed in sCJD patients compared to healthy controls. The same miRNAs behave differently in patients diagnosed with Alzheimer’s disease (AD) emphasizing their specificity for sCJD. Additionally, the identified miRNA cluster correlates with sCJD severity in a longitudinal observation of sCJD patients.
Norsworthy et al. provide interesting and novel insights into pathology of human prion disease outside the central nervous system. The findings are of interest for both neuroscientists and physicians with an expertise in neurodegenerative diseases. The experimental data in the manuscript provides solid evidence for miRNA alterations in sCJD but the authors’ claim for a diagnostic assay based on blood miRNA is weak and needs to be substantiated by more analyses.

Major issue:

- While the dysregulation of let-7i-5p, miR-16-5p, miR-93-5p, miR-106b-3p, let-7d-3p is clear in aggregated data, there seems to be a large overlap in miRNA expression between individual sCJD and healthy controls. Can the authors perform more statistical analyses and present sensitivity and specificity of individual and aggregated miRNAs for the diagnosis of sCJD? Youden indices could be calculated as optimal cut-off points. An area under the receiver operating characteristics curve (AUC ROC) could be calculated to demonstrate the diagnostic performance. If possible, the authors could calculate improvement of diagnostic accuracy between sCJD and AD based on demographic/clinical features in the presence or absence of results from miRNA assay.

Minor issues:
- Can the authors add a supplementary table of target identification strategy as described on page 12, last paragraph? Top miRNA targets could be displayed with validated assays, number of publications, expression levels in whole blood from GTEx database
- It would be interesting to know the proportions of probable / definitive sCJD cases in the discovery and validation cohorts. Did probable sCJD cases undergo RT-QuIC testing? Does the picture change significantly if only definitive sCJD cases or definitive + probable with positive RT-QuIC are considered?

Karl Frontzek


Reviewer #3 (Remarks to the Author):

Norsworthy and collaborators studied blood microRNA (miRNA) expression in sCJD in search of novel molecular signatures that might help disease diagnosis and monitoring of clinical progression or provide unique insights into the pathobiology of the disease.
The authors initially studied 57 patients and 50 healthy controls and subsequently validated their findings in 29 sCJD patients and 30 controls. Besides, they investigated 30 patients with AD.
They found a significant downregulation of 3 miRNA with a concomitant upregulation of four of their mRNA target in sCJD, but not in AD and controls.

The manuscript is well-written, technically sound, addresses an important topic, and includes original data of potential interest. However, the findings appear preliminary in relation to the specific aims of the study. A diagnostic test for sCJD must be able to distinguish the disease from other forms of rapidly progressive dementia. In this study, the authors only compared sCJD with healthy controls and AD patients. Moreover, no data on the diagnostic accuracy of the test in the differential diagnosis between AD and sCJD is provided. From the data presented in fig. 3, it seems that the specificity will likely be relatively low, given the significant overlap between the two groups.
Similarly, the data on the correlation with disease progression is based on a limited number of heterogeneous patients (MV and VV patients usually have a significantly different rate of progression and, therefore, should be ideally kept separate in this kind of analysis). Moreover, data from different groups have shown that plasma levels of tau correlate with disease progression in sCJD. Thus, it would be important to show how these novel molecular signatures perform in comparison to tau levels.
Finally, no data are presented regarding the possible meaning of the findings for the understanding of the pathobiology of sCJD.

Additional points the authors should consider:

1) The statement in the abstract indicating that no blood test is available for sCJD is misleading (see my comment above about tau, for example). The authors should consider rephrasing it.
2) Using TarBase, the authors identified six mRNA, which might be targeted by the three downregulated miRNA. However, only 4 out of 6 of them have an increased expression in the blood of sCJD patients. It is, therefore, unclear why the authors used all six mRNA for the pathway enrichment analysis instead of only the 4 showing increased expression. Moreover, it seems that they used a lower threshold for mRNA than for miRNA and that 2 of the four mRNA have e mean increased expression lower than 1.5 times.
3) As also acknowledged by the authors in the discussion (line 273), the analysis of brain tissue might help the comprehension of the pathogenetic role, if any, of the identified miRNA and their target mRNA. As the first step in this direction, the authors may compare the mRNA targets of the identified miRNA in brain and blood using TarBase.
4) More clinical details, such as symptoms at onset and during progression, results of neurological examinations, and laboratory and instrumental findings, should be provided (perhaps in a supplementary table) for the 15 patients that were followed longitudinally. The authors may also want to explain why only one MM case (the most common genotype in affected patients) was included in the analysis.
5) Reference 6 is self-referential and does reflect originality and recognition by the expert scientific community of the available literature on the topic.

**Author rebuttal, first version:**

**Reviewer #1 (Remarks to the Author):**

In this study, a signature of 5 altered microRNAs (miRNAs) in blood was identified in a cohort of sporadic Creutzfeldt-Jakob disease (sCJD) patients using small RNA-sequencing. The decreased abundance of 3/5 miRNAs were further replicated in an independent sCJD cohort using quantitative PCR (qPCR). Additionally, increased abundance in blood of 4 target genes of the miRNA signature was also detected in the sCJD patients. By measuring the expression of the miRNA signature in Alzheimer’s disease (AD) patients it was also revealed that this signature can discriminate between sCJD and AD. Finally, by longitudinally measuring miRNA expression in a third sCJD cohort, the rate of decline for the 3 miRNAs was compared to the rate of sCJD disease progression. This study strongly benefits from a careful experimental design and relatively large numbers of individual samples, which can be difficult to achieve for sCJD due to the rarity of this disorder.
It is of interest to others in the prion field as it is the first to provide a robust analysis of circulating miRNAs in a human prion disease, although one study of serum miRNAs in chronic wasting disease, a prion disease of deer was recently published (<https://doi.org/10.1038/s41598-019-56249-6>). Statistical analysis is appropriate and for the most part there is sufficient detail for a researcher to reproduce the work.

*We would like to thank the reviewer for her/his comments, especially in acknowledging the difficulties inherent in working with patient samples from rare disorders. We are pleased the reviewer finds our work carefully designed and our results interesting.*

*We are very grateful to the reviewer for highlighting the recent paper by Slota et al. We have read this paper with great interest. The work by Slota et al, which was published after the initial submission of our manuscript on 31^st^ October 2019, is indeed highly relevant to our work because it identified candidate miRNA biomarkers in elk infected with prion disease. We have now referred to this paper in both the introduction and discussion section of the revised manuscript (pages 4 and 10 respectively). We also cite the interesting work from Ludwig et al. in the discussion section of the revised manuscript (page 12).*

Specific comments:

1. In this manuscript miRNA profiling is performed in whole blood rather than serum or plasma. This has some advantages such as it is less prone to the variations caused by sample manipulation, results are not biased by cell lysis and it is more amenable to normalization using commonly used housekeeping genes.

However, there are also some disadvantages that have not been discussed in the manuscript. Extremely high levels of a number of miRNAs are associated with red blood cells (RBCs) for example. When using a direct ‘count’ based strategy such as NGS this means that changes in less abundant miRNAs tend to be masked, thus concealing disease specific patterns. This should be mentioned in relation to the relatively limited correlation found with other studies of altered miRNA abundance in prion diseases.

*We agree with the reviewer that profiling miRNA in whole blood rather than serum has some advantages and also brings some disadvantages. We thank the reviewer for suggesting we discuss this important aspect of our study. As suggested by the reviewer, we now discuss these aspects in the revised manuscript (page 12).*

A second disadvantage that is not mentioned in the manuscript is miRNAs are expressed in varying amounts in the different cell types found in blood. (e.g. <https://doi.org/10.1016/j.gpb.2019.09.004>). The authors should comment on biological variation in cell counts in relation to disease and disease progression and how that may affect the miRNA profiles obtained. Are the authors able to associate cell counts with miRNA abundance? Are cell counts available for the patients in the study and can this data be used in a normalization step for the data? If so could the authors reanalyze the data to account for, for example, RBC contribution to the profiles. It is possible that more disease related profiles could be determined by performing some further normalization steps.

*We thank the reviewer for making this very important point. To address this comment, we used four approaches in order to test whether disease status would impact cell counts.*

1. *First, a literature search. The question of possible alteration to blood counts has been investigated previously. In 2009, a study led by S. Prusiner reported that neither White Blood Cell (WBC) composition nor the amount of cell-surface PrP^C^ molecules was altered in sCJD patients (Choi et al., 2009; reference 22). This work suggests that sCJD cases cannot be distinguished by their altered blood counts. To our knowledge, the effect of sCJD disease progression on cell counts has not been investigated.*
2. *We then wanted to confirm this was the case in our sample set. To this end, we intended to write to physicians who had referred the patients included in our study to the National Prion Clinic. The aim was to collect information about full blood counts. Unfortunately, this attempt was abruptly stopped because of the outbreak of COVID-19. However, we were able to access full blood counts for 13 sCJD patients (8 females; 5 males) aged between 48 and 75 years old. For each sCJD patient, 9 fractions were reported: white cell count, red cell count, haemoglobin, platelet, neutrophils, lymphocytes, monocytes, eosinophils and basophils. Four patients had marginally elevated values for one or more cell type (i.e. outside the normal range). The results are presented in the table below:*

| sCJD patients (8 female, 5 male) | Sampling age | White cell count (x10^9^/L) | Red cell count (x10^12^/L) | Haemoglobin  (g/L) |
| --- | --- | --- | --- | --- |
| Normal range | N/A | 3.0 - 10.0 | 3.95 - 5.15 | 115 - 155 |
| sCJD Range | 48-75 | 4.48-11.68 | 4.03-5.58 | 114-171 |
| Mean | 60 | 7.28 | 4.56 | 135 |
| Standard deviation | 9 | 2.07 | 0.43 | 15 |

| **sCJD patients (8 female, 5 male)** | **Platelets (x10^9^/L)** | **Neutrophils (x10^9^/L)** | **Lymphocytes (x10^9^/L)** | **Monocytes (x10^9^/L)** | **Eosinophils (x10^9^/L)** | **Basophils (x10^9^/L)** |
| --- | --- | --- | --- | --- | --- | --- |
| **Normal range** | 150 - 400 | 2.0 - 7.5 | 1.2 - 3.65 | 0.2 - 1.0 | 0.0 - 0.4 | 0.0 - 0.1 |
| **sCJD Range** | 215-440 | 2.53-9.1 | 1.30-2.57 | 0.31-0.82 | 0.03-0.31 | 0.01-0.07 |
| **Mean** | 298 | 4.65 | 1.87 | 0.58 | 0.15 | 0.03 |
| **Standard deviation** | 69 | 1.92 | 0.36 | 0.15 | 0.08 | 0.02 |

*These blood cell counts, although not from patients included in our cohorts, support the published work by Choi et al., in that data from sCJD considerably overlaps normal ranges. These results suggest that sCJD cases cannot be distinguished from healthy individuals by their blood counts.*

3. *Third, and importantly, we used DNA methylation markers to estimate cell proportions in blood samples from 93 individuals (40 controls; 53 sCJD) out of the 105 in our discovery cohort. To do so, DNA was extracted from blood stored in EDTA tubes and whole genome DNA methylation profiles were generated using Illumina arrays (450K or EPIC). Because EDTA tubes do not preserve RNA, it was crucially important to match sampling dates, so that Pax Tubes (used for RNA) and EDTA tubes (used for DNA) were collected on the same day. This is to ensure that the cell proportion estimate obtained from DNA methylation markers would be as accurately representing the cell count as possible.*

*After quality control, 432,263 methylation sites from all 93 samples were analyzed through established pipelines using the Houseman algorithm which allows cell-type deconvolution from DNA methylation profiles. This algorithm is routinely used in blood methylome study and Epigenome Wide Association Studies (EWAS). The method developed by Houseman et al.* (<https://doi.org/10.1186/1471-2105-13-86>) *in 2012 utilizes DNA methylation as a surrogate estimate of immune cell proportions. This algorithm is a reference-based deconvolution algorithm that takes advantage of known cell-type specific Differentially Methylated Regions (DMRs). Using this method, we estimated the relative proportion of CD8 T cells, CD4 T cells, Natural Killer (NK) cells, B cells, Monocytes and Granulocytes in each of the 93 samples. We found no statistical difference in cell type proportions between sCJD and controls as illustrated by the table below:*

| **Cell type** | **Mean sCJD cell count (%)** | **Mean control cell count (%)** | **P value** | **Statistical test** |
| --- | --- | --- | --- | --- |
| CD8 T cells | 1.87x10^-15^ (Median) | 3.04x10^-15^ (Median) | 0.102 | Mann-Whitney |
| CD4 T cells | 10.9 | 13.3 | 0.141 | T Test |
| NK cells | 0.7 (Median) | 0.8 (Median) | 0.880 | Mann-Whitney |
| B Cells | 2.5 | 3.3 | 0.066 | T Test |
| Monocytes | 6.2 | 6.1 | 0.940 | T Test |
| Granulocytes | 76.7 | 72.7 | 0.091 | T Test |

4. *Finally, as suggested by the reviewer, we used the cell composition estimations generated by the DNA methylation analysis to associate cell counts with miRNA abundance. The table attached (Appendix Table 1, see page 9 of this document) shows that sCJD cases cannot be distinguished from controls by their altered blood counts.*

*Altogether, the results from these four lines of investigation led us to believe that normalization of the sequencing data using cell counts is not required here. We anticipate that further normalization steps based on cell composition of blood samples will not improve the number of miRNAs in the signature we identified. For all these reasons, we did not reanalyse the data presented in the manuscript. The effect of disease progression, however, was not investigated.*

*Thanks to the reviewer’s comment, we have now added a sentence about cell counts in the results section (page 5) of the revised manuscript. Given that the DNA methylation data would require considerable explanation and methodology in the revised paper, for reasons of space we have not added it to the revised manuscript, but we would be prepared to do this at the request of the editorial team.*

Accordingly miR-16-5p, identified in this study as a significant disease-associated miRNA, is extremely highly expressed in red blood cells. The authors comment on the importance of this miRNA to prion disease in reference to studies on infected brain tissue. Is it possible that altered abundance in the blood is unrelated to the mechanism by which it is deregulated in neurons or neurodegeneration specifically?

*This is another good point made by the reviewer. Indeed miR-16-5p is abundant in blood and we should have discussed the possibility that its altered abundance in that tissue might be independent of what is happening in brain. We have now incorporated this comment and have modified the discussion section of the revised manuscript (page 10) accordingly.*

2. It is mentioned that 1 million reads was the cut-off in these analyses. Given that a small number of miRNAs make up the majority of read counts in blood this is likely a suboptimal number of reads for this type of study. If some samples resulted in significantly more reads than others perhaps the authors could comment on, or provide an analysis of some of the lower abundance miRNAs if they can be identified in these samples.

*We thank the reviewer for this comment. We have carefully looked at our discovery data and identified 36 samples with more than 1.5 million reads (14 cases and 22 controls). Based on the reviewer’s comment we have re-run the analysis on this subset of samples to take lower abundance miRNAs into account. This added another 30 miRNAs to the list of miRNAs tested for differential expression between sCJD and controls. None of them was found to be significantly differentially expressed. We have now included this data in Supplementary Data 2, as suggested by the reviewer.*

3. The very rapid decline in sCJD would likely mean that these patients would only correlate to AD patients very late in their generally much longer disease course. When comparing AD and sCJD samples confirm that the disease stage is equivalent.

*Thank you for this comment. The reviewer is quite correct to point out that these are disorders with distinct trajectories of disease severity. A comparison of disease severity is not straightforward as this is measured in different ways for the two diseases. A range of rating scales were measured in both sets of patients, but these were different for each disease e.g. MMSE for AD, and more bespoke functional scales in prion diseases. We could also look at time from symptom onset.*

*We have concluded that the patient groups were substantially overlapping in disease severity, but a more formal statistical comparison is difficult. Samples were selected from patients at the first diagnostic assessment, so match the clinical situation of a first encounter with a specialist physician whose priority is to establish a diagnosis.*

*We also considered our correlative and longitudinal data. Given that the selected miRNAs do not correlate with disease severity measured by MRC Scale score in sCJD and that the selected miRNAs do not correlate with disease severity measured by MMSE score in AD, this is probably not a significant confounding issue.*

4. In figure 4 a significant relationship was observed between the change in relative miRNA expression per day and %loss of function per day. However, it appears from the graphs that a single sample with the fastest decline in miRNA expression and fastest rate of disease progression is driving statistical significance, with the remaining samples showing little correlation between miRNA expression and disease progression. The claim that miRNA expression associates with disease progression should be strengthened by addressing if this sample is an outlier (is statistical significance retained if the sample is removed?) or if any confounding factors could have influenced miRNA abundance in this sample. It is also possible that the relationship is non-linear.

*We thank the reviewer for making this point. As a result of this comment we undertook to analyse an extra 13 samples taken from 6 sCJD cases (each with 2 or 3 samples). We took extra care to select patients with medium and fast disease progression since those would populate the right-hand side of the graphs and increase statistical power. miRNA expression was profiled using the same qPCR assay as before. Adding more samples abolished the significance of the initial findings for let-7i, miR-16-5p and miR-93. In other words, we do not have evidence that miRNA expression correlates with disease progression.*

*We have amended the revised manuscript accordingly. We believe that these findings are important for the community because longitudinal samples sets are precious and rare, and we have therefore kept these results as main Figure 4.*

Additionally, no information was provided regarding the timing of the longitudinal sampling (i.e. how long between each sampling). This should be included to provide more information about how the rate of miRNA decline was calculated, perhaps as a Supplemental table or figure.

*We apologise for not providing this information. Thanks to the reviewer’s comment we have included the number of samples, and interval between sampling, for patients in the longitudinal cohort in the revised version of the manuscript (Supplementary Data 3). Moreover, we also have included the median sampling interval in the revised Table 1.*

5. A recent publication that showed increased let-7i-5p abundance in serum from elk with chronic wasting disease and decreased miR-16-5p abundance in serum from scrapie infected hamsters was not referenced. See:
Slota JA, Medina SJ, Klassen M, et al. Identification of circulating microRNA signatures as potential biomarkers in the serum of elk infected with chronic wasting disease. Sci Rep. 2019;9(1):19705. Published 2019 Dec 23. doi:10.1038/s41598-019-56249-6

In this manuscript greater correlation between serum miRNA profiles in a prion disease and neurodegeneration (AD) was reported than seen here. Do the authors think that this may be evidence of masking of disease related patterns by the inclusion of red blood cell miRNAs in the analysis in this case?

*We are very grateful to the reviewer for highlighting the recent paper by Slota et al. In this paper, which was published after the initial submission of our manuscript in October 2019, the authors report a greater correlation between serum miRNA profiles and neurodegeneration (AD) than we report here. As suggested by the reviewer, one possible explanation for this discrepancy could be that the protocol used to extract RNA from blood does not totally eliminate red blood cells, and that remaining traces might contribute to masking disease related patterns. Although the first step of the protocol we used to extract the RNA removes red blood cells, we cannot exclude the possibility that some traces might be left in samples.*

*In order to respond to this comment, we generated a list of miRNAs known to be affected by hemolysis and compared their levels with those in our discovery study. One possibility is that disease status, or the handling of blood samples, could lead to hemolysis. If this was the case, we would expect to see altered levels of miRNAs known to be affected by hemolysis in our dataset. The results presented in Appendix Table 2 (page 10 of this document) show that those miRNAs, reported to be strongly affected by hemolysis by Kirschner et al. in 2013 (reference 51), are not consistently altered in our dataset.*

*Furthermore, using both sequencing and qPCR, we also report that hsa-miR-484 levels remain unchanged between sCJD and controls. This is important because hsa-miR-484 levels are known to increase in hemolysed serum (Kirschner et al., 2013; reference 51). Altogether, these suggest that our results are unlikely to be affected by red blood cell contamination. We have discussed this in our revised manuscript (page 12).*

6. Clarify that the RIN numbers were those of total RNA rather than the small RNA fraction.

*We thank the reviewer for highlighting this imprecision. RIN numbers are indeed those of total RNA. We now have made this clear in both discussion and methods sections (pages 12 and 14 respectively) of the revised manuscript.*

7. Why were the read counts expressed as FPKM as this is generally used in RNA Seq to normalize paired end reads with the varying lengths of transcripts. Here the experiment is designed to capture only reads of mature fully processed miRNAs 15-25bp.

*We thank the reviewer for highlighting this. We used Cufflinks for read quantification and normalization, because no miRNA-seq specific quantification tools were available within Partek Flow at the time of analysis. As Cufflinks was originally developed for RNA-seq, it outputs counts in FPKM which is why these units were used in our manuscript. As pointed out by the reviewer, for miRNA sequencing there is indeed no need for length-based normalization because single-end reads of short length are captured. FPKM, which removes the feature-length and library-size effects, is therefore analogous to TPM (transcripts per million).*

8. Clarify the method of normalization of the read data – for example, global mean normalization scaling?

*Thank you to the referee for asking us to clarify this. In the manuscript we used Cufflinks to normalize the reads. More specifically, the default option ‘total hits normalization’ was used. With this option Cufflinks counts all fragments, including those not compatible with any reference transcript, towards the number of mapped hits used in the FPKM denominator. An alternative normalization option within Cufflinks, is ‘upper quartile normalization’. We have reanalysed our data using this option and found that the results remain unchanged*. *Thanks to the reviewer’s comment we have now included this information in the methods section of the revised manuscript (page 15).*

9. It is encouraged to make datasets such as this publicly available by submitting to databases such as NCBI’s GEO.

*We couldn’t agree more with the reviewer. Less than a week after our manuscript was submitted, we made the datasets publicly available by submitting them to NCBI’s GEO. The accession number GSE140069 has been added to the methods section of the manuscript (page 17).*

10. It would be beneficial to see the inclusion of PCA plots based on analysis of the total data. This is a useful way for visual quality control. This could be included as a figure/supplementary data to clearly illustrate the relationship between samples and to highlight any outlier samples.

*We thank the reviewer for making this important suggestion. We have now included the PCA plot of the discovery study as a new Supplementary Fig.1.*

*Finally, we would like to bring to the attention of the reviewer the fact that the numbers (fold change and p values) described on page 5, and in Supplementary Data 1, of the manuscript have been amended. This is because, during the revision process, two control samples were removed from the discovery cohort. This was done because during our data audit it became apparent that two healthy controls had been bled twice and both samples inadvertently included. As these were not truly independent samples the second samples were removed. The discovery cohort now comprises 105 samples originating from 105 different individuals. The analyses have been re-run and all results presented in Table 1, Figure 1, Supplementary Data 1 and Supplementary Table 1 have been updated accordingly. There were no changes to our conclusions based on this re-analysis. We deeply apologise for any confusion caused and have highlighted these changes on page 5.*

Appendix Table 1

*Correlation coefficients and p values for Pearson correlation of log transformed normalised read counts for DE miRNAs with methylation data derived cell type proportions.*

| **DE miRNA** | **hsa-let-7i-5p** | | **hsa-miR-93-5p** | | **hsa-miR-16-5p** | | **hsa-let-7d-3p** | | **hsa-miR-106b-3p** | |
| --- | --- | --- | --- | --- | --- | --- | --- | --- | --- | --- |
| Cell fraction | Correlation coefficient | p value | Correlation coefficient | p value | Correlation coefficient | p value | Correlation coefficient | p value | Correlation coefficient | p value |
| CD8 T cells | 0.0609 | 0.5729 | 0.0406 | 0.7073 | 0.1522 | 0.1568 | 0.0237 | 0.8267 | 0.0749 | 0.4878 |
| CD4 T cells | 0.1628 | 0.1297 | 0.1582 | 0.1410 | 0.2074 | 0.0525 | -0.1383 | 0.1988 | 0.1921 | 0.0730 |
| Natural Killer cells | 0.1351 | 0.2094 | 0.0440 | 0.6838 | 0.1484 | 0.1676 | -0.0742 | 0.4920 | 0.1360 | 0.2065 |
| B cells | 0.2838 | 0.0074 | 0.2387 | 0.0251 | 0.2934 | 0.0055 | -0.1921 | 0.0730 | 0.2054 | 0.0548 |
| Monocytes | 0.1109 | 0.3035 | 0.1670 | 0.1200 | 0.1216 | 0.2591 | 0.0285 | 0.7924 | 0.1486 | 0.1672 |
| Granulocytes | -0.2445 | 0.0217 | -0.2158 | 0.0435 | -0.3070 | 0.0036 | 0.1387 | 0.1975 | -0.2634 | 0.0132 |

**Appendix Table 2**

*Combined list of miRNAs whose expression has been shown to be altered in hemolysed blood samples. Those with a change in expression >10 fold are highlighted in red. Highlighted in green are the three miRNAs significantly altered in sCJD, and hsa-miR-484, which we used in our study as a non-DE control miRNA.*

| **miRNAs** | **Direction of effect in hemolysed samples** | **Reference (Author, year)** | **Fold change in our sequencing dataset; sCJD vs controls** |
| --- | --- | --- | --- |
| hsa-let-7f-5p | Up and down | Kirschner *et al* 2013 | -1.865264377 |
| hsa-let-7g-5p | Up and down | Kirschner *et al* 2013 | -2.359214693 |
| hsa-miR-148b | Up and down | Kirschner *et al* 2013 | Not analysed |
| hsa-miR-197-3p | Up and down | Kirschner *et al* 2013 | 1.405829874 |
| hsa-miR-30e-3p | Up and down | Kirschner *et al* 2013 | -1.082434886 |
| hsa-let-7b-5p | UP >10X | Kirschner *et al* 2013 | 1.025657975 |
| hsa-miR-140-3p | UP >10X | Kirschner *et al* 2013 | -1.140313361 |
| hsa-miR-16-5p | UP >10X | Kirschner *et al* 2013 | -2.758476581 |
| hsa-miR-19a | UP >10X | Kirschner *et al* 2013 | Not analysed |
| hsa-miR-625-3p | UP >10X | Kirschner *et al* 2013 | 1.069779568 |
| hsa-miR-15a-5p | UP >10X | Kirschner *et al* 2013 | -4.05673285 |
| hsa-miR-192-5p | UP >10X | Kirschner *et al* 2013 | -1.338084132 |
| hsa-miR-195 | UP >10X | Kirschner *et al* 2013 | Not analysed |
| hsa-miR-20b | UP >10X | Kirschner *et al* 2013 | Not analysed |
| hsa-miR-25-3p | UP >10X | Kirschner *et al* 2013 | -1.415856288 |
| hsa-miR-122 | UP | Kirschner *et al* 2013 | Not analysed |
| hsa-miR-20a | UP | Kirschner *et al* 2013 | Not analysed |
| hsa-miR-210-3p | UP | Kirschner *et al* 2013 | -1.82499071 |
| hsa-miR-320 | UP | Kirschner *et al* 2013 | Not analysed |
| hsa-miR-425-5p | UP | Kirschner *et al* 2013 | 1.125991148 |
| hsa-miR-454-5p | UP | Kirschner *et al* 2013 | -1.104398252 |
| hsa-miR-484 | UP | Kirschner *et al* 2013 | 1.128333765 |
| hsa-miR486-3p | UP | Kirschner *et al* 2013 | Not analysed |
| hsa-miR-140-3p | UP | Kirschner *et al* 2013 | -1.140313361 |
| hsa-miR-101 | UP | Kirschner *et al* 2013 | Not analysed |
| hsa-miR-103 | UP | Kirschner *et al* 2013 | Not analysed |
| hsa-miR-106a | UP | Kirschner *et al* 2013 | Not analysed |
| hsa-miR-106b-3p | UP | Kirschner *et al* 2013 | -1.739473091 |
| hsa-miR-126 | UP | Kirschner *et al* 2013 | Not analysed |
| hsa-miR-133a | UP | Kirschner *et al* 2013 | Not analysed |
| hsa-miR-144 | UP | Kirschner *et al* 2013 | Not analysed |
| hsa-miR-15b-5p | UP | Kirschner *et al* 2013 | -1.972080018 |
| hsa-miR17 | UP | Kirschner *et al* 2013 | Not analysed |
| hsa-miR-185-5p | UP | Kirschner *et al* 2013 | 1.149052648 |
| hsa-miR-186-5p | UP | Kirschner *et al* 2013 | -1.019288055 |
| hsa-miR-19b-3p | UP | Kirschner *et al* 2013 | -1.543178199 |
| hsa-miR-21 | UP | Kirschner *et al* 2013 | Not analysed |
| hsa-miR-26b-3p | UP | Kirschner *et al* 2013 | 1.126641356 |
| hsa-miR-29c | UP | Kirschner *et al* 2013 | Not analysed |
| hsa-miR-30a-5p | UP | Kirschner *et al* 2013 | Not analysed |
| hsa-miR-30d-5p | UP | Kirschner *et al* 2013 | -1.103273399 |
| hsa-miR-3208 | UP | Kirschner *et al* 2013 | Not analysed |
| hsa-miR-324 | UP | Kirschner *et al* 2013 | -1.016735483 |
| hsa-miR-331-5p | UP | Kirschner *et al* 2013 | Not analysed |
| hsa-miR-340 | UP | Kirschner *et al* 2013 | Not analysed |
| hsa-miR-378 | UP | Kirschner *et al* 2013 | Not analysed |
| hsa-miR-485-5p | UP | Kirschner *et al* 2013 | Not analysed |
| hsa-miR-532 | UP | Kirschner *et al* 2013 | 1.099709393 |
| hsa-miR-574 | UP | Kirschner *et al* 2013 | 1.193954355 |
| hsa-miR-590-5p | UP | Kirschner *et al* 2013 | Not analysed |
| hsa-miR-652-3p | UP | Kirschner *et al* 2013 | -1.229320697 |
| hsa-miR-660 | UP | Kirschner *et al* 2013 | Not analysed |
| hsa-miR-92a | UP | Kirschner *et al* 2013 | Not analysed |
| hsa-miR-93-5p | UP | Kirschner *et al* 2013 | -2.337568953 |
| hsa-miR-99b-5p | UP | Kirschner *et al* 2013 | 1.426991395 |
| hsa-miR-125a | UP | Kirschner *et al* 2013 | 1.486817343 |
| hsa-miR-126 | UP | Kirschner *et al* 2013 | Not analysed |
| hsa-miR-132 | UP | Kirschner *et al* 2013 | Not analysed |
| hsa-miR-139-5p | UP | Kirschner *et al* 2013 | Not analysed |
| hsa-miR-143 | UP | Kirschner *et al* 2013 | Not analysed |
| hsa-miR-145 | UP | Kirschner *et al* 2013 | Not analysed |
| hsa-miR-146b | UP | Kirschner *et al* 2013 | Not analysed |
| hsa-miR-150-5p | UP | Kirschner *et al* 2013 | 1.038610713 |
| hsa-miR-15b | UP | Kirschner *et al* 2013 | -1.972080018 |
| hsa-miR-18a | UP | Kirschner *et al* 2013 | 1.093670009 |
| hsa-miR-18b | UP | Kirschner *et al* 2013 | Not analysed |
| hsa-miR-191 | UP | Kirschner *et al* 2013 | Not analysed |
| hsa-miR-193b | UP | Kirschner *et al* 2013 | Not analysed |
| hsa-miR-194 | UP | Kirschner *et al* 2013 | Not analysed |
| hsa-miR-199a-3p | UP | Kirschner *et al* 2013 | Not analysed |
| hsa-miR-23a | UP | Kirschner *et al* 2013 | Not analysed |
| hsa-miR-29a | UP | Kirschner *et al* 2013 | Not analysed |
| hsa-miR-30b-5p | UP | Kirschner *et al* 2013 | 1.322003901 |
| hsa-miR-323-3p | UP | Kirschner *et al* 2013 | Not analysed |
| hsa-miR-335 | UP | Kirschner *et al* 2013 | Not analysed |
| hsa-miR-338-5p | UP | Kirschner *et al* 2013 | Not analysed |
| hsa-miR-339-3p | UP | Kirschner *et al* 2013 | -1.012660951 |
| hsa-miR-342-3p | UP | Kirschner *et al* 2013 | -1.001691903 |
| hsa-miR-374 | UP | Kirschner *et al* 2013 | Not analysed |
| hsa-miR-485-3p | UP | Kirschner *et al* 2013 | Not analysed |
| hsa-miR-493 | UP | Kirschner *et al* 2013 | Not analysed |
| hsa-miR-532-3p | UP | Kirschner *et al* 2013 | 1.099709393 |
| hsa-miR-601 | UP | Kirschner *et al* 2013 | Not analysed |
| hsa-miR-661 | UP | Kirschner *et al* 2013 | Not analysed |
| hsa-miR-942-5p | UP | Kirschner *et al* 2013 | -1.399911537 |
| hsa-let7a-5p | Not affected | Kirschner *et al* 2013 | -1.48 |
| hsa-miR-127 | Not affected | Kirschner *et al* 2013 | Not analysed |
| hsa-miR-1274A | Not affected | Kirschner *et al* 2013 | Not analysed |
| hsa-miR-1274B | Not affected | Kirschner *et al* 2013 | Not analysed |
| hsa-miR-130b-3p | Not affected | Kirschner *et al* 2013 | 1.092499213 |
| hsa-miR-142-5p | Not affected | Kirschner *et al* 2013 | -2.192819529 |
| hsa-miR-146a | Not affected | Kirschner *et al* 2013 | Not analysed |
| hsa-miR-221 | Not affected | Kirschner *et al* 2013 | Not analysed |
| hsa-miR-27b | Not affected | Kirschner *et al* 2013 | Not analysed |
| hsa-miR-324-5p | Not affected | Kirschner *et al* 2013 | -1.084982835 |
| hsa-miR-744-5p | Not affected | Kirschner *et al* 2013 | 1.184332633 |
| hsa-miR-23a | Not affected | Blondal *et al* 2013 | Not analysed |
| hsa-miR-30b-5p | affected | Myklebust *et al* 2019^a^ | 1.322003901 |
| hsa-miR-20a-5p | affected | Myklebust *et al* 2019 | Not analysed |

^a^<https://doi.org/10.3389/fgene.2019.00463>

**Reviewer #2 (Remarks to the Author):**

The paper by Norsworthy and co-workers investigates the role of blood microRNAs (miRNAs) in patients diagnosed with sporadic Creutzfeldt-Jakob disease (sCJD) and their feasibility as blood-based diagnostic assay. The clinical diagnosis of sCJD is usually based on the combination of a neuropsychiatric syndrome and presence of pathologically aggregated prion protein in an aggregation assay done on the patient’s cerebrospinal fluid. Obtaining CSF by lumbar puncture is not without risks such as post-puncture headaches, bleeding, infection. Here, a blood-based assay for the diagnosis of sCJD could represent a substantial improvement. Additionally, majority of sCJD research has been focused on the central nervous system while changes in peripheral organs are widely unclear.
The paper is well-written and adequately powered considering the rare incidence of sCJD. The authors show that a subset of miRNAs is differentially expressed in sCJD patients compared to healthy controls. The same miRNAs behave differently in patients diagnosed with Alzheimer’s disease (AD) emphasizing their specificity for sCJD. Additionally, the identified miRNA cluster correlates with sCJD severity in a longitudinal observation of sCJD patients.
Norsworthy et al. provide interesting and novel insights into pathology of human prion disease outside the central nervous system. The findings are of interest for both neuroscientists and physicians with an expertise in neurodegenerative diseases.

*We are very grateful to the reviewer for his comments and finding our work interesting, for noting the relevance of our work beyond the prion field, and for acknowledging the efforts we took in carefully designing the experiments and meeting their statistical requirements.*

The experimental data in the manuscript provides solid evidence for miRNA alterations in sCJD but the authors’ claim for a diagnostic assay based on blood miRNA is weak and needs to be substantiated by more analyses.

*We have taken this point into account and thanks to the comments made by the reviewer we believe we have substantiated our claim and strengthened our conclusions. In particular, we have done this by responding to his major issue and generating ROC curves and calculating Youden indices. We now provide these new data in a new Figure 5 in the revised manuscript. Our new analyses revealed that measuring the expression of three miRNAs (let-7i-5p, miR-16-5p, and miR-93-5p) in patients with sCJD achieves potentially useful diagnostic accuracy in whole blood. Our new results also show that the expression levels of these three miRNAs offer a possible avenue for a differential diagnostic to discriminate sCJD from AD, using blood samples.*

Major issue:

- While the dysregulation of let-7i-5p, miR-16-5p, miR-93-5p, miR-106b-3p, let-7d-3p is clear in aggregated data, there seems to be a large overlap in miRNA expression between individual sCJD and healthy controls. Can the authors perform more statistical analyses and present sensitivity and specificity of individual and aggregated miRNAs for the diagnosis of sCJD? Youden indices could be calculated as optimal cut-off points. An area under the receiver operating characteristics curve (AUC ROC) could be calculated to demonstrate the diagnostic performance. If possible, the authors could calculate improvement of diagnostic accuracy between sCJD and AD based on demographic/clinical features in the presence or absence of results from miRNA assay.

*We are grateful to the reviewer for suggesting this. We have performed more analyses and now provide sensitivity and specificity for each individual miRNA (new Supplementary Table 4). We have done this for each of the following comparisons:*

- *sCJD versus controls in the discovery cohort;*
- *sCJD versus controls in the replication cohort;*
- *AD versus controls;*
- *sCJD versus AD.*

*We found that combining (or aggregating) the three miRNAs together performs better than each single one individually for all but the “sCJD versus AD” comparison. In this case, let-7i-5p performs slightly better than all 3 miRNAs combined (AUC 0.934 and 0.924 respectively).*

*The revised manuscript now contains all this information. We have added Figure 5 and Supplementary Fig. 8-10 and Table 4. To ensure that these results were not unique for normalization to RNU6-2, we also performed these analyses for each comparison using each of the normalizing genes, namely SNORD42B, SNORD95, and SNORD72 (new Supplementary Fig. 8-10). These did not demonstrate any different trend compared to the one presented in Figure 5.*

*Thanks to the reviewer’s comment, we have significantly improved the manuscript and strengthened our conclusions. We find that miRNA expression is altered in blood from sCJD patients. And that these changes are a signal in the periphery rather than just a consequence of brain degeneration. Therefore, it might be that blood miRNA expression could potentially be used as a non-invasive marker to help differentiate between prion infection and other neurodegenerative diseases in humans. In the discussion section of the revised manuscript, we do however point out that diagnostic assays solely based on miRNA expression would be unlikely to provide the same level of accuracy as assays which rely on specific detection of PrP^Sc^ such as PMCA or RT-QuIC. Future work will help establish whether miRNA profiles used in combination with other biomarkers, and demographic and clinical features, improve diagnostic accuracy in neurodegenerative diseases.*

Minor issues:
- Can the authors add a supplementary table of target identification strategy as described on page 12, last paragraph? Top miRNA targets could be displayed with validated assays, number of publications, expression levels in whole blood from GTEx database

*We thank the reviewer for his comment and apologise for not including this information in our initial submission. The revised manuscript now contains this table (New Supplementary Table 3).*

- It would be interesting to know the proportions of probable / definitive sCJD cases in the discovery and validation cohorts. Did probable sCJD cases undergo RT-QuIC testing? Does the picture change significantly if only definitive sCJD cases or definitive + probable with positive RT-QuIC are considered?

Karl Frontzek

*This is an important point and we thank the reviewer for raising it. We are now providing information about probable sCJD cases in the revised Table 1. We report the following:*

- *There are 3 probable sCJD cases in the discovery cohort (out of 57 cases) and 1 probable sCJD case (out of 29 cases) in the replication cohort. In the longitudinal cohort, there are 8 probable sCJD cases out of 21 patients.*
- *Only one probable sCJD case underwent RT-QuIC testing.*
- *We have highlighted the probable sCJD cases in the discovery cohort and shown that these are not outliers (new Supplementary Fig. 1).*
- *When analysing the sequencing data from definite sCJD cases only, our results remain unaffected. Results are presented in new Supplementary Table 2.*

*Future work might allow us to consider cases with positive and negative RT-QuIC results.*

*Finally, we would like to bring to the attention of the reviewer the fact that the numbers (fold change and p values) described on page 5, and in Supplementary Data 1, of the manuscript have been amended. This is because, during the revision process, two control samples were removed from the discovery cohort. This was done because during our data audit it became apparent that two healthy controls had been bled twice and both samples inadvertently included. As these were not truly independent samples the second samples were removed. The discovery cohort now comprises 105 samples originating from 105 different individuals. The analyses have been re-run and all results presented in Table 1, Figure 1, Supplementary Data 1 and Supplementary Table 1 have been updated accordingly. There were no changes to our conclusions based on this re-analysis. We deeply apologise for any confusion caused and have highlighted these changes on page 5.*

Reviewer #3 (Remarks to the Author):

Norsworthy and collaborators studied blood microRNA (miRNA) expression in sCJD in search of novel molecular signatures that might help disease diagnosis and monitoring of clinical progression or provide unique insights into the pathobiology of the disease.
The authors initially studied 57 patients and 50 healthy controls and subsequently validated their findings in 29 sCJD patients and 30 controls. Besides, they investigated 30 patients with AD. They found a significant downregulation of 3 miRNA with a concomitant upregulation of four of their mRNA target in sCJD, but not in AD and controls.
The manuscript is well-written, technically sound, addresses an important topic, and includes original data of potential interest.

*We thank the reviewer for his/her comments and for finding our work of interest.*

However, the findings appear preliminary in relation to the specific aims of the study. A diagnostic test for sCJD must be able to distinguish the disease from other forms of rapidly progressive dementia. In this study, the authors only compared sCJD with healthy controls and AD patients. Moreover, no data on the diagnostic accuracy of the test in the differential diagnosis between AD and sCJD is provided. From the data presented in fig. 3, it seems that the specificity will likely be relatively low, given the significant overlap between the two groups.

*We thank the reviewer for this comment. We have substantiated our claim and strengthened our conclusions. In particular, in generating ROC curves and calculating Youden indices we now provide these new data in new Figure 5 in the revised manuscript. This analysis led us to provide sensitivity and specificity data for the differential diagnosis between sCJD and AD using miRNA expression in whole blood samples.*

*More specifically, we found both let-7i-5p on its own, and all 3 miRNAs combined perform very well in differentiating sCJD from AD cases (AUC 0.934 and 0.924 respectively).*

*We also performed the same analysis for each of the following comparisons:*

- *sCJD versus controls in the discovery cohort;*
- *sCJD versus controls in the replication cohort;*
- *AD versus controls.*

*We found that aggregating the 3 miRNAs together performs better than each single one individually (new Figure 5, new Supplementary Table 4 and new Supplementary Fig 8-10).*

*Thanks to the reviewer’s comment, we have strengthened our conclusions. These new results are important because they identify non-protein blood markers that could, in combination with existing diagnostic methods, improve sCJD disease management. They are also important because they highlight that changes in miRNA expression in the periphery might be more than just a consequence of brain degeneration. We discuss this in the discussion section of the revised manuscript (page 13).*

Similarly, the data on the correlation with disease progression is based on a limited number of heterogeneous patients (MV and VV patients usually have a significantly different rate of progression and, therefore, should be ideally kept separate in this kind of analysis. Moreover, data from different groups have shown that plasma levels of tau correlate with disease progression in sCJD. Thus, it would be important to show how these novel molecular signatures perform in comparison to tau levels.

*We thank the reviewer for this very important point. Thanks to his/her comment we undertook to analyse an extra 13 samples taken from 6 sCJD cases (with 2-3 samples per case). We took extra care to select patients with medium and fast disease progression since those would populate the right-hand side of the graphs. miRNA expression was profiled using the same qPCR assay as before. Adding more samples removed the significance of the initial findings for let-7i and miR-16-5p and miR-93. In other words, miRNA expression does not correlate with disease progression.*

*We have amended the title, abstract, introduction, results and discussion sections of the revised manuscript accordingly. We believe that these findings are important for the community because longitudinal sample collections are rare and have therefore decided to keep these results as main Figure 4.*

Moreover, data from different groups have shown that plasma levels of tau correlate with disease progression in sCJD. Thus, it would be important to show how these novel molecular signatures perform in comparison to tau levels. Finally, no data are presented regarding the possible meaning of the findings for the understanding of the pathobiology of sCJD.

*We agree with the reviewer that correlating tau levels with the expression levels of small non coding RNAs in blood would be of great interest. We hope that future work will provide responses to this question.*

*Similarly, we believe that our data support the existence of a signal of sCJD in the periphery. Whether this signal is related to the pathobiology of the disease is of great interest, but in a definitive study would require experimental manipulation of miRNA levels and is therefore beyond the scope of this manuscript.*

Additional points the authors should consider:

1) The statement in the abstract indicating that no blood test is available for sCJD is misleading (see my comment above about tau, for example). The authors should consider rephrasing it.

*We thank the reviewer for this comment and apologise for this misleading sentence. Following the reviewer’s suggestion, we have rephrased the sentence to “clinical blood tests” in the revised manuscript (page 2, Abstract).*

2) Using TarBase, the authors identified six mRNA, which might be targeted by the three downregulated miRNA. However, only 4 out of 6 of them have an increased expression in the blood of sCJD patients. It is, therefore, unclear why the authors used all six mRNA for the pathway enrichment analysis instead of only the 4 showing increased expression.

*We thank the reviewer for highlighting this. We should have indeed included the four miRNAs showing increased expression in the pathway enrichment analysis. We apologise for this oversight. We have corrected this and performed the analysis using only those four miRNAs. No significant results remained after p value adjustment. We included this information in the results section of the revised manuscript (page 7).*

Moreover, it seems that they used a lower threshold for mRNA than for miRNA and that 2 of the four mRNA have e mean increased expression lower than 1.5 times.

*We apologise for the lack of clarity in describing the thresholds. The 1.5 fold change was used as a visual delineator to guide the eye in Figure 1a. We have now made this clear in the revised legend.*

3) As also acknowledged by the authors in the discussion (line 273), the analysis of brain tissue might help the comprehension of the pathogenetic role, if any, of the identified miRNA and their target mRNA. As the first step in this direction, the authors may compare the mRNA targets of the identified miRNA in brain and blood using TarBase.

*We thank the reviewer for making this suggestion. We used GTEx to compare the expression of the miRNA targets in brain and blood. As shown below, we did not find any consensus. The CCND3 transcript is more abundant in blood whilst ZFP36 and CDNK1A levels are comparable in blood and brain tissues. The case of NAP1L1, target of let-7i-5p, is interesting because its transcript levels appear to be higher in brain regions than in blood. This is especially true in the spinal cord. The data used for the analyses described in this manuscript were obtained from: the GTEx Portal on 04/24/20. Graphs are presented on page 19 of this document.*

*We would like to point out that this information presents the expression profiles of miRNAs targets in healthy tissues. The impact of sCJD on these profiles remains unknown. Our results suggest that changes in expression profiles of miRNA are a signal in the periphery rather than just a consequence of brain degeneration. Future work will help in deciphering the mechanisms involved in these changes in the periphery and understanding their contribution to brain pathogenicity.* *Thanks to the reviewer’s comment we have included this in the discussion section of the revised manuscript (page 13).*

4) More clinical details, such as symptoms at onset and during progression, results of neurological examinations, and laboratory and instrumental findings, should be provided (perhaps in a supplementary table) for the 15 patients that were followed longitudinally. The authors may also want to explain why only one MM case (the most common genotype in affected patients) was included in the analysis.

*We thank the reviewer for this suggestion, and apologise for not providing this information earlier. We have taken up the suggestion and have included as much clinical data as we could gather in new Supplementary Data 3. Moreover, we also have included 6 more sCJD cases in our longitudinal cohort (new Figure 4), two of those with the MM genotype. Although MM is the most common genotype in sCJD cases, it is also the one that is most strongly associated with speed of decline. Therefore, collecting samples from MM individuals that could be included in a longitudinal study is more difficult than from patients with slower progressing disease (mostly with MV and VV genotypes).*

5) Reference 6 is self-referential and does reflect originality and recognition by the expert scientific community of the available literature on the topic.

*We thank the reviewer for highlighting this and have now replaced this reference with Parchi et al, Ann Neurol, 1999 in the revised manuscript.*

*Finally, we would like to bring to the attention of the reviewer the fact that the numbers (fold change and p values) described on page 5, and in Supplementary Data 1, of the manuscript have been amended. This is because, during the revision process, two control samples were removed from the discovery cohort. This was done because during our data audit it became apparent that two healthy controls had been bled twice and both samples inadvertently included. As these were not truly independent samples the second samples were removed. The discovery cohort now comprises 105 samples originating from 105 different individuals. The analyses have been re-run and all results presented in Table 1, Figure 1, Supplementary Data 1 and Supplementary Table 1 have been updated accordingly. There were no changes to our conclusions based on this re-analysis. We deeply apologise for any confusion caused and have highlighted these changes on page 5*

**Reviewer comments, second version:**

Reviewer #1 (Remarks to the Author):

Thank-you very much for the detailed responses that have satisfactorily answered my queries to the authors.


Reviewer #2 (Remarks to the Author):

The authors have sufficiently addressed the reviewer's suggestions resulting in a substantially revised manuscript, I recommend it for publication.


Reviewer #3 (Remarks to the Author):

The authors addressed most of my concerns. I have the following further suggestion:
Without comparison with the performance of other established blood biomarkers such as tau or NfL and, above all, without an analysis of a real clinical cohort of patients with suspected CJD, the current results on the diagnostic potential of blood miRNA biomarkers remain preliminary. The authors should consider adding a sentence underlying this limitation.

**Author rebuttal, second version:**

**Reviewer #1 (Remarks to the Author)**:

Thank-you very much for the detailed responses that have satisfactorily answered my queries to the authors.

*We are glad we have answered the reviewer’s comments, thank you.*

**Reviewer #2 (Remarks to the Author)**:

The authors have sufficiently addressed the reviewer's suggestions resulting in a substantially revised manuscript, I recommend it for publication.

*We thank the reviewer for his/her comments and for recommending our work for publication.*

**Reviewer #3 (Remarks to the Author)**:

The authors addressed most of my concerns. I have the following further suggestion:
Without comparison with the performance of other established blood biomarkers such as tau or NfL and, above all, without an analysis of a real clinical cohort of patients with suspected CJD, the current results on the diagnostic potential of blood miRNA biomarkers remain preliminary. The authors should consider adding a sentence underlying this limitation.

*We are grateful for the reviewer’s suggestion and have included this point in our revised discussion (page 12).*

**Reviewer comments, third version:**

**Author rebuttal, third version:**
